# A genetic disorder reveals a hematopoietic stem cell regulatory network co-opted in leukemia

**DOI:** 10.1038/s41590-022-01370-4

**Published:** 2022-12-15

**Authors:** Richard A. Voit, Liming Tao, Fulong Yu, Liam D. Cato, Blake Cohen, Travis J. Fleming, Mateusz Antoszewski, Xiaotian Liao, Claudia Fiorini, Satish K. Nandakumar, Lara Wahlster, Kristian Teichert, Aviv Regev, Vijay G. Sankaran

**Affiliations:** 1 Division of Hematology/Oncology, Boston Children’s Hospital, Harvard Medical School, Boston, MA USA; 2 Department of Pediatric Oncology, Dana-Farber Cancer Institute, Harvard Medical School, Boston, MA USA; 3 Broad Institute of MIT and Harvard, Cambridge, MA USA; 4 Howard Hughes Medical Institute, Chevy Chase, MD USA; 5 Department of Biology, Massachusetts Institute of Technology, Cambridge, MA USA; 6 Harvard Stem Cell Institute, Cambridge, MA USA; 7 Present Address: Genentech, South San Francisco, CA USA; 8 Present Address: Department of Cell Biology, Albert Einstein College of Medicine, Albert Einstein Cancer Center, Ruth L. and David S. Gottesman Institute for Stem Cell Research and Regenerative Medicine, Bronx, NY USA

**Keywords:** Haematopoietic stem cells, Gene regulation in immune cells, Haematopoiesis, Primary immunodeficiency disorders, Leukaemia

## Abstract

The molecular regulation of human hematopoietic stem cell (HSC) maintenance is therapeutically important, but limitations in experimental systems and interspecies variation have constrained our knowledge of this process. Here, we have studied a rare genetic disorder due to *MECOM* haploinsufficiency, characterized by an early-onset absence of HSCs in vivo. By generating a faithful model of this disorder in primary human HSCs and coupling functional studies with integrative single-cell genomic analyses, we uncover a key transcriptional network involving hundreds of genes that is required for HSC maintenance. Through our analyses, we nominate cooperating transcriptional regulators and identify how MECOM prevents the CTCF-dependent genome reorganization that occurs as HSCs differentiate. We show that this transcriptional network is co-opted in high-risk leukemias, thereby enabling these cancers to acquire stem cell properties. Collectively, we illuminate a regulatory network necessary for HSC self-renewal through the study of a rare experiment of nature.

## Abstract (web summary)

Modeling a rare bone marrow failure disorder due to haploinsufficiency for the MECOM transcription factor identifies a human hematopoietic stem cell regulatory network, which is co-opted by high-risk leukemias.

## Main

HSCs lie at the apex of the hierarchical process of hematopoiesis and rely on transcriptional regulators to coordinate self-renewal and lineage commitment to enable effective and continuous blood cell production^1^. Perturbations of HSC maintenance or differentiation result in a spectrum of hematopoietic consequences, ranging from bone marrow failure to leukemia^2^. Despite the importance of HSCs in human health and the therapeutic opportunities that could arise from being able to better manipulate these cells, the precise regulatory networks that maintain these cells remain poorly understood.

Recently, loss-of-function mutations in myelodysplastic syndrome (MDS) and ecotropic virus integration site-1 (EVI1) complex locus (*MECOM*) have been identified that lead to a severe neonatal bone marrow failure syndrome^3–5^. Haploinsufficiency of *MECOM* leads to near complete loss of HSCs within the first months of life, suggesting an important and dosage-dependent role in early hematopoiesis. In mice, different *Mecom* isoforms have distinct hematopoietic functions^6–8,9,10^, but the ability of *Mecom* haploinsufficient mice to maintain sufficient hematopoietic output stands in sharp contrast to the profound and highly penetrant HSC loss observed in patients with *MECOM* haploinsufficiency, irrespective of which isoform is impacted. This interspecies variation suggests that the clinical observations in *MECOM* haploinsufficiency may provide a unique opportunity to better understand human HSC regulation.

*MECOM* overexpression has been reported in ~10% of adult and pediatric acute myeloid leukemias (AMLs) and is associated with a particularly poor prognosis^11^. Despite the potential mechanisms of MECOM activity that have been suggested from studies in AML cell lines^12–15^, the holistic functions of MECOM that enable effective human HSC maintenance and drive leukemia remain enigmatic. Here, inspired by in vivo observations from patients who are *MECOM* haploinsufficient, we have modeled this disorder by genome editing of primary human CD34^+^ hematopoietic stem and progenitor cells (HSPCs). Through integrative single-cell genomic analyses in this model, we define fundamental transcriptional regulatory circuits necessary for human HSC maintenance. Finally, we demonstrate that this same HSC transcriptional regulatory network is co-opted in AML, thereby conferring stem cell features and a poor prognosis.

## Results

### MECOM loss impairs HSC function in vitro and in vivo

**Fig. 1:**
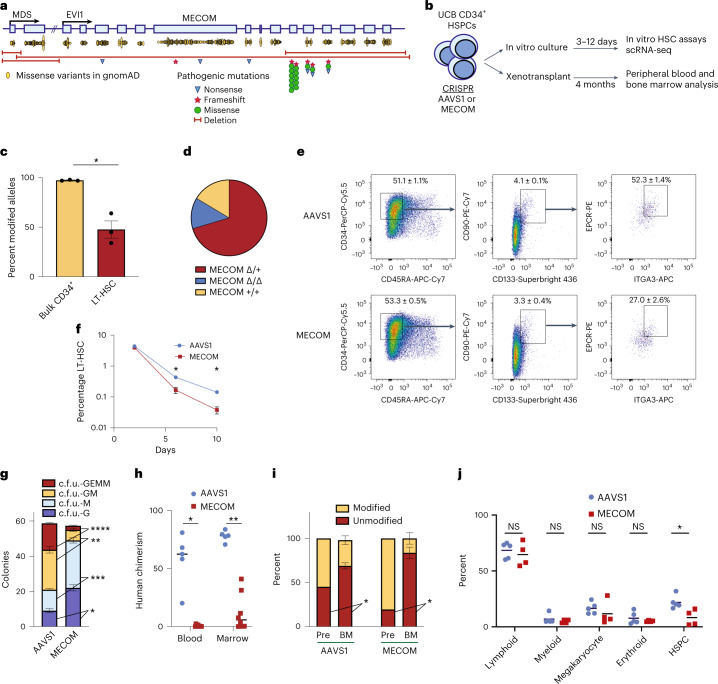
Generating a faithful model of *MECOM* haploinsufficiency and HSC loss. **a**, Schematic of the *MECOM* locus displaying two coding exons of *MDS* (MDS 2–3) and 15 coding exons of *EVI1* (EVI1 2–16). Yellow ovals represent frequency and location of missense variants from individuals in the gnomAD database. Pathogenic variants from patients with bone marrow failure include nonsense (blue triangles), frameshift (red stars) and missense mutations (green circles) as well as large deletions (red bars). **b**, Experimental outline of *MECOM* editing and downstream analysis in human UCB-derived HSCs. **c**, Bar graph of the frequency of modified *MECOM* alleles in bulk CD34^+^ human HSPCs or in LT-HSCs. HSPCs that underwent CRISPR editing were cultured in HSC medium containing UM171. On day 6 after editing, genotyping by PCR and Sanger sequencing was performed on bulk HSPCs or LT-HSCs sorted by fluorescence-activated cell sorting (FACS). Mean of three independent experiments is plotted and error bars show s.e.m. Two-sided Student’s *t*-test was used. *P = 0.0048. **d**, Pie chart showing the proportion of *MECOM* genotypes in single-cell LT-HSCs following *MECOM* perturbation. Overall, 189 single-cell LT-HSCs were genotyped using single-cell genomic DNA sequencing and classified as either wild-type (MECOM^+/+^, yellow), heterozygous edited (MECOM^Δ/+^, red) or homozygous edited (MECOM^Δ/Δ^, blue). **e**,**f**, Phenotypic analysis of LT-HSCs after *MECOM* editing. **e**, Gating strategy to identify phenotypic LT-HSCs after CRISPR editing of *AAVS1* or *MECOM*. LT-HSCs are defined as CD34^+^CD45RA^-^CD90^+^CD133^+^EPCR^+^ITGA3^+^. Mean (± s.e.m.) in the highlighted gates on day 6 after CRISPR editing is shown (*n* = 3) and the total LT-HSC percentage is the product of the frequencies in each gate shown. **f**, Time course showing that *MECOM* editing leads to progressive loss of phenotypic LT-HSCs in vitro. The *x* axis displays days after CRISPR editing and the *y* axis displays the percent of live cells in the LT-HSC gate as defined above. Mean of three independent experiments is plotted and error bars show s.e.m. Error bars that are shorter than the size of the symbol in the AAVS1 samples have been omitted for clarity. Two-sided Student’s *t*-test was used. *P = 0.003. **g**, Stacked bar plots of colony-forming assay comparing *MECOM*-edited UCB-derived CD34^+^ HSPCs (*n* = 3) to *AAVS1*-edited controls (*n* = 3). Three days after CRISPR perturbation, cells were plated in methylcellulose and colonies were counted after 14 d. *MECOM* editing leads to reduced formation of multipotent c.f.u. GEMM and bipotent c.f.u. GM progenitor colonies and an increase in unipotent colonies. Mean colony number is plotted and error bars show s.e.m. Two-sided Student’s *t*-test was used. *P = 3.3 × 10^−3^, **P = 1.4 × 10^−3^, ***P = 7.8 × 10^−4^, ****P = 4.5 × 10^−5^. **h**, Analysis of peripheral blood and bone marrow of mice at week 16 following xenotransplantation of *MECOM*-edited (*n* = 8) and *AAVS1*-edited (*n* = 5) HSPCs. Mean is indicated by black line and each data point represents one mouse. Two-sided Student’s *t*-test was used. *P = 5 × 10^−6^, **P = 2 × 10^−6^. **i**, Comparison of edited allele frequency following xenotransplantation. *MECOM*-edited cells in bone marrow after xenotransplantation are enriched for unmodified alleles as detected by next-generation sequencing (NGS), revealing a selective engraftment disadvantage of HSPCs with *MECOM* edits. Pre, pre-transplant; BM, bone marrow. Mice with human chimerism >2% are included in this analysis (AAVS1, 5 of 5 mice; MECOM, 4 of 8 mice) Mean is plotted and error bars show s.e.m. Two-sided Student’s *t*-test was used. *P = 0.02. **j**, Subpopulation analysis of human cells in mouse BM after xenotransplantation. Cell populations were identified by the following surface markers: lymphoid, CD45^+^CD19^+^; myeloid, CD45^+^CD11b^+^; megakaryocyte, CD45^+^CD41a^+^; erythroid, CD235a^+^; and HSPC, CD34^+^. Only mice with human chimerism >2% were included in the analysis (AAVS1, 5 of 5 mice; MECOM, 4 of 8 mice). Mean is indicated by black lines and each data point represents one mouse. Two-sided Student’s *t*-test was used. NS, not significant, *P = 0.01. Source data

**Extended Data Fig. 1:**
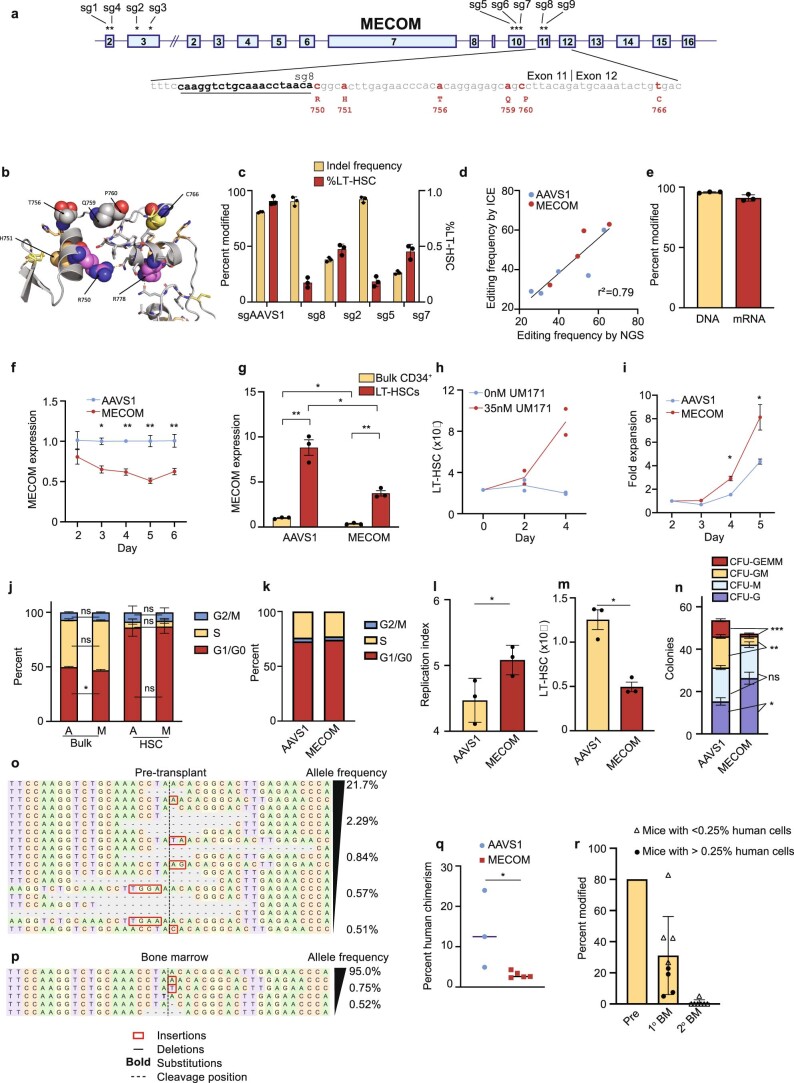
Modeling *MECOM* haploinsufficiency in human CD34^+^ HSPCs. **(a)** Schematic of the *MECOM* locus annotated with the location of sgRNAs (sg1-sg9) tested for efficiency of *MECOM* editing. The binding site of sg8 (underlined) which is used in subsequent studies is shown, and clinical mutations annotated with amino acid number that have been described in *MECOM* haploinsufficient bone marrow failure (red) are indicated. **(b)** Predicted partial protein structure of the MECOM zinc finger domain with mutated residues shown as spheres. These mutations are expected to disrupt the structure of the zinc finger, either through abrogation of Zn coordination (H751, C766) or tethering between the ZnF (R750, R778). **(c)** Percent modified alleles (left y-axis) and percent LT-HSCs of total live cells (right y-axis) after CRISPR editing of primary human CD34^+^ HSPCs. Editing efficiency was detected at 72 hours after RNP delivery of Cas9 and sgRNA by nucleofection and percent of live cells that remained in the LT-HSC gate was evaluated on day 6. LT-HSCs are defined by the following immunophenotype: CD34^+^CD45RA^−^CD90^+^CD133^+^EPCR^+^ITGA3^+^. sg2, sg5, sg7, sg8 are sgRNAs targeting MECOM as described in Extended Data Fig. 1a. *n* = 3 biologically independent samples. Mean is plotted and error bars show s.e.m. **(d)** Comparison of Sanger sequencing followed by ICE analysis and Next Generation Sequencing (NGS) for the detection of CRISPR edits. *AAVS1* (blue) and *MECOM* (red) edited samples were analyzed by ICE and NGS in parallel. **(e)** *MECOM* editing in human CD34^+^ HSPCs after RNP delivery by nucleofection. Editing frequency was detected at 48 hours by Sanger sequencing of genomic DNA. Transcription of edited *MECOM* alleles was determined by cDNA synthesis followed by Sanger sequencing of RNA from bulk HSPCs at 48 hours. *n* = 3 biologically independent samples. Mean is plotted and error bars show s.e.m. **(f)** *MECOM* expression following CRISPR editing. *MECOM* expression (normalized to *GAPDH*) in bulk HSPCs was detected by qRT-PCR (*n* = 3 AAVS1, *n* = 9 MECOM; three biologically independent experiments) and was normalized to expression in the AAVS1-edited sample on the same day. Mean is plotted and error bars show s.e.m. Two-sided Student *t*-test used. *P = 1.7e-3, **P = 2.5e-4. **(g)** *MECOM* expression in LT-HSCs. *MECOM* expression (normalized to *GAPDH*) was detected by qRT-PCR (*n* = 3 per group; three biologically independent experiments) in bulk CD34^+^ HSPCs and in LT-HSCs sorted on day 3 after CRISPR editing. Mean is plotted and error bars show s.e.m. Two-sided Student *t*-test used. *P = 5.1e-3, **P = 8.3e-4. **(h)** Expansion of LT-HSCs in culture. HSPCs were cultured in the presence (*n* = 2) or absence (*n* = 2) of the HSC self-renewal agonist UM171. Percent of LT-HSCs was determined by FACS as in Fig. 1e and was used to calculate the total LT-HSC number. Cells were supplemented with fresh media every 2 days. **(i)** Expansion time course of bulk CD34^+^ HSPCs following CRISPR editing. HSPCs were thawed into HSC media containing 35 nM UM171 and underwent CRISPR editing 24 hours later. Cells were counted daily by trypan blue exclusion starting on day 2 after CRISPR editing and media was added to maintain equal confluency. *n* = 3 per group. Mean is plotted and error bars show s.e.m. Error bars that are shorter than the size of the symbols have been omitted for clarity. Two-sided Student *t*-test used. *P = 5e-3. **(j)** Stacked bar graph of cell cycle status of bulk HSPCs and HSC (HSC: CD34^+^CD45RA^−^CD90^+^CD133^+^) as determined by Edu incorporation and 7-AAD staining. On day 5 after CRISPR editing, cells were incubated with Edu for 2 hours, then fixed and permeabilized prior to 7-AAD and cell surface staining. *AAVS1*-edited (A) and *MECOM*-edited (M) samples, were compared by the proportion of cells in G0/G1 (Edu^−^/2n DNA content), S (Edu^+^), or M (Edu^−^/>2n DNA content) in bulk CD34^+^ cells or CD34^+^CD45RA^−^CD90^+^ HSCs. *n* = 3 per group. Mean is plotted and error bars show s.e.m. Two-sided Student *t*-test used. *P = 8.1e-3. **(k)** Stacked bar graph of cell cycle status of LT-HSCs as determined by transcriptional signatures of single-cell LT-HSCs. UCB CD34^+^ underwent CRISPR perturbation of *MECOM* or *AAVS1* and were maintained in HSC media. On day 4 after editing, LT-HSCs were sorted and 10x scRNA sequencing was performed. There was no difference in cell cycle state in LT-HSCs following *AAVS1* or *MECOM* editing. **(l)** Analysis of cell expansion following CRISPR editing. *AAVS1* or *MECOM* edited HSPCs were labeled with CFSE and successive generations of cell divisions were determined by CFSE signal intensity on day 5 which was used to calculate the replication index, showing the total number of divided cells/cells that underwent at least one division. Mean of three independent experiments is plotted and error bars show s.e.m. Two-sided Student *t*-test used. *P = 5e-2. **(m)** Total number of LT-HSCs following MECOM editing. Primary human CD34 + HSPCs underwent CRISPR editing on day 1 after thawing and were cultured in HSC media containing UM171 which was changed every 2 days. On day 6 after editing, the percentage of immunophenotypic LT-HSCs determined by flow cytometry, and the total cell number determined by trypan blue exclusion were used to calculate the total number of LT-HSCs in culture. Mean of three independent experiments is plotted and error bars show s.e.m. Two-sided Student *t*-test used. *P = 4.7e-3. **(n)** Stacked bar plots of colony-forming assay comparing *MECOM* edited HSPCs derived from peripherally mobilized CD34^+^ cells from healthy adult donors. (*n* = 6) to *AAVS1*-edited controls (*n* = 3). CFU-GEMM, colony-forming unit (CFU) granulocyte erythroid macrophage megakaryocyte; CFU-GM, CFU granulocyte macrophage; CFU-M, CFU macrophage; CFU-G, CFU granulocyte. Mean colony number is plotted and error bars show s.e.m. Two-sided Student *t*-test used. *P = 3.9e-2, **P = 2.5e-4, ***P = 1.7e-5, ns=not significant. **(o-p)** NGS of *MECOM* in human HSPCs following CRISPR editing, prior to xenotransplantation **(o)**, and after harvest from bone marrow at 16 weeks of one representative mouse **(p)**. Sequences present at frequencies >0.5% are displayed. **(q)** Analysis of bone marrow of mice at week 16 following transplantation of *MECOM*-edited (*n* = 5) and *AAVS1*-edited (*n* = 3) adult HSPCs. Mean is indicated by black line and each data point represents one mouse. Two-sided Student *t*-test used. *P = 3.8e-2. **(r)** Analysis of the *MECOM* locus of human cells harvested from mice following primary or secondary xenotransplantation. Half of the primary recipient mice (4/8) had human chimerism >0.25% (circles) and the other half had chimerism <0.25% (triangles) but had human *MECOM* sequences that were detectable by PCR. All of the secondary recipients had human chimerism <0.25% but had human *MECOM* sequences that were detectable by PCR. Source data

Monoallelic mutations spanning the coding sequence of *MECOM* have been reported in at least 31 individuals with severe, early-onset neonatal bone marrow failure (Fig. 1a, Supplementary Table 1 and Extended Data Fig. 1a,b)^3–5^. The paucity of HSCs associated with *MECOM* haploinsufficiency prevents the mechanistic study of primary patient samples^4^, so we sought to develop a model to study *MECOM* haploinsufficiency in primary human cells by disrupting *MECOM* via CRISPR editing in CD34^+^ HSPCs purified from umbilical cord blood (UCB) samples of healthy newborns (Fig. 1b and Extended Data Fig. 1a,c,d). We achieved editing at >80% of alleles in the bulk CD34^+^ population, but the subpopulation of CD34^+^CD45RA^−^CD90^+^CD133^+^EPCR^+^ITGA3^+^ phenotypic long-term HSCs (LT-HSCs)^16^ displayed 48% editing of *MECOM* alleles (Fig. 1c), allowing for predominantly heterozygous edits in the LT-HSC compartment. Genotyping of single LT-HSCs following *MECOM* perturbation confirmed that 70% were heterozygous for *MECOM* edits (Fig. 1d), although this is likely an underestimation given that allelic dropout is common in single-cell genotyping^17^. These edits were transcribed to messenger RNA, but reduced transcript levels, possibly due to nonsense-mediated decay^18^ (Extended Data Fig. 1e–g).

*MECOM*-edited human HSPCs underwent 1.9-fold higher expansion over 5 d in culture conditions that promote HSC maintenance^19^ (Extended Data Fig. 1h,i), consistent with previous observations of differentiation and expansion of HSCs after MECOM loss^8^. *MECOM* perturbation was associated with a decrease in the proportion of bulk cells in G0/G1 on day 5, but no difference in the cell cycle states of HSCs (Extended Data Fig. 1j). Most HSCs remained in G0/G1 and the majority of LT-HSCs had G0/G1 transcriptional signatures (Extended Data Fig. 1k), as previously reported^20^. *MECOM* editing resulted in more frequent cell divisions (Extended Data Fig. 1l) and a significant reduction in the absolute number of LT-HSCs (Extended Data Fig. 1m), with a 3.7-fold reduction by day 10 after editing (Fig. 1e,f). We observed a 6.4-fold reduction in multipotent colony-forming unit (c.f.u.) granulocyte erythroid macrophage megakaryocyte (GEMM) colonies and a 3.8-fold reduction in bipotent c.f.u. granulocyte macrophage (GM) colonies, along with increases in more differentiated unipotential c.f.u. granulocyte (G) and c.f.u. macrophage (M) colonies (Fig. 1g). There was a similar loss of multipotent and bipotent progenitor colonies derived from adult HSPCs following *MECOM* editing (Extended Data Fig. 1n), validating the importance of this factor across developmental stages.

Next, we performed non-irradiated xenotransplantation of edited HSPCs into immunodeficient and Kit-mutant (Methods) mice to assess how MECOM loss impacts human HSCs in vivo^21^. *MECOM*-edited HSPCs engrafted in only half of the transplanted animals with significantly lower human chimerism in the peripheral blood and bone marrow compared to *AAVS1*-edited controls (Fig. 1h). When we compared the edited allele frequency of cells collected from the bone marrow at 16 weeks with the cells before transplant, we found a fivefold enrichment of the unmodified *MECOM* allele (Fig. 1i and Extended Data Fig. 1o,p), consistent with selection occurring against *MECOM*-edited HSCs. In the mouse bone marrow, there was a 2.7-fold reduction in human CD34^+^ HSPCs in the *MECOM*-edited samples, but no detectable differences in engrafted lymphoid, erythroid, megakaryocytic or monocytic lineages (Fig. 1j). Similarly, we found significant reduction in human chimerism following primary xenotransplantation of adult HSPCs following *MECOM* editing (Extended Data Fig. 1q). When we performed secondary xenotransplantation of UCB HSPCs, we observed moderate secondary engraftment of *AAVS1*-edited cells (two of five mice), but no detectable secondary engraftment of *MECOM*-edited cells (zero of eight mice). To more sensitively assay for the presence of human cells in the secondary transplant recipients, we PCR-amplified human *MECOM* from all bone marrow samples. Sequencing revealed 100% wild-type *MECOM* in seven of eight secondary recipients and 95% in the remaining mouse (Extended Data Fig. 1r). This near complete absence of *MECOM* edits in serially repopulating LT-HSCs is consistent with the profound HSC loss observed in patients with *MECOM* haploinsufficiency. In summary, our model of *MECOM* haploinsufficiency reveals that MECOM is required for maintenance of LT-HSC in vitro and in vivo and enables us to capture LT-HSCs before their complete loss to directly study MECOM function.

### Single-cell profiling reveals HSC loss after MECOM disruption

**Fig. 2:**
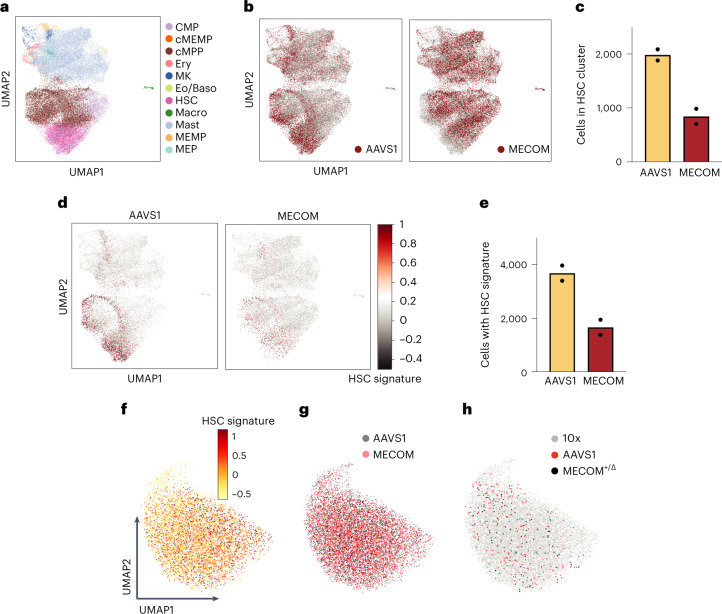
Loss of transcriptional HSCs after MECOM perturbation. **a**, Uniform Manifold Approximation and Projection (UMAP) plot and cell type clustering of human HSCs after CRISPR editing. UCB CD34^+^ cells underwent CRISPR editing and were sorted 3 d later for CD34^+^CD45RA^-^CD90^+^ HSCs followed by scRNA-seq. Cells were clustered by transcriptional signatures using Celltypist^22^. CMP, common myeloid progenitor; MEMP, megakaryocyte-erythroid-mast cell progenitor; cMEMP, cycling MEMP; MEP, megakaryocyte-erythroid progenitor; cMPP, cycling multipotent progenitor; Ery, early erythroid progenitor; MK, early megakaryocyte progenitor; Eo/baso, eosinophil/basophil progenitor; Macro, macrophage progenitor; Mast, mast cell progenitor. **b**, UMAP plot of CD34^+^CD45RA^-^CD90^+^ HSCs stratified by CRISPR edits, showing the depletion of HSCs following *MECOM* perturbation. *AAVS1*-edited sample highlighted in red (left). *MECOM*-edited sample highlighted in red (right). Each sample is the combination of two biological replicates. **c**, Bar graph showing the number of cells in the HSC cluster in *AAVS1*- and *MECOM*-edited samples. Mean is plotted and each of two biological replicates is shown. Total number of cells profiled in each group was 19,375 (AAVS1) and 19,821 (MECOM). **d**, UMAP plot of CD34^+^CD45RA^−^CD90^+^ HSCs following CRISPR editing (*AAVS1*-edited (left), *MECOM*-edited (right)), colored according to expression of HSC signature (*CD34*, *HLF* and *CRHBP*). **e**, Bar graph showing the number of cells expressing the three-gene HSC signature. An HSC signature score >0.5 indicates high expression. Mean is plotted and each of two biological replicates is shown. Total number of cells profiled in each group was 19,375 (AAVS1) and 19,821 (MECOM). **f**–**h**, UMAP plots of CD34^+^CD45RA^−^CD90^+^CD133^+^EPCR^+^ITGA3^+^ LT-HSCs following CRISPR editing, indicating enrichment of the HSC signature as determined by scRNA-seq using the 10x Genomics platform (**f**), overlap of *AAVS1*-edited and the *MECOM*-edited cells, sequenced using the 10x Genomics platform (**g**) and distribution of cells with monoallelic *MECOM* edits determined by G&T sequencing by SmartSeq2, compared to *AAVS1*-edited cells and LT-HSCs from **f** (**h**). Source data

**Extended Data Fig. 2:**
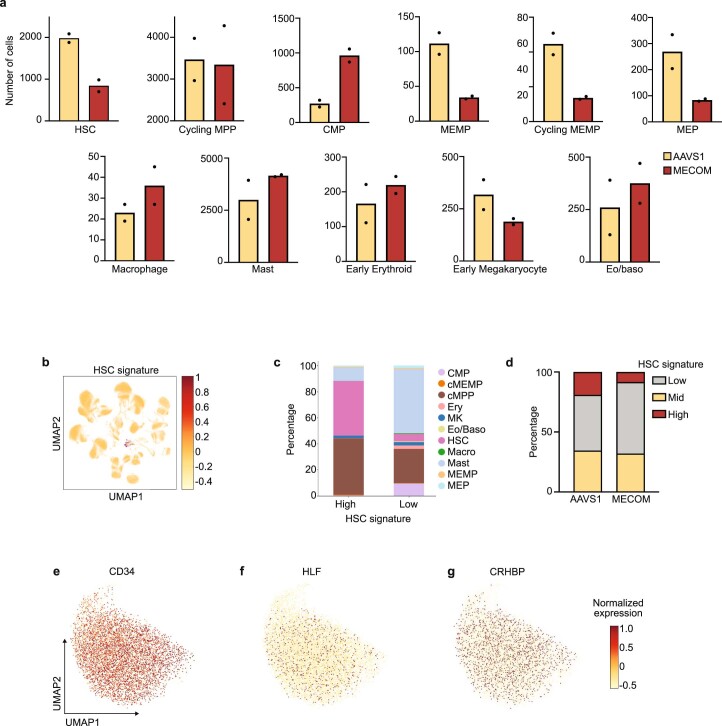
Single-cell RNA sequencing of LT-HSCs after MECOM editing. **(a)** Bar graphs showing the number of cells in each of the 11 hematopoietic cell clusters identified by single cell RNA sequencing of CD34^+^CD45RA^−^CD90^+^ HSCs following AAVS1 (yellow) or MECOM (red) editing. Mean is plotted and each of two biological replicates is shown. Total number of cells profiled in each group: AAVS1 – 19,375, MECOM – 19,821. **(b)** Uniform manifold approximation and projection (UMAP) plot of 263,828 single cells from human umbilical cord blood, colored according to HSC signature (*CD34*, *HLF*, *CRHBP*). **(c)** Transcriptional identities of cells stratified by HSC signature score. HSC signature score was calculated for CD34^+^CD45RA^−^CD90^+^ HSCs from Fig. 2d. Cells were grouped into high HSC signature score (>0.5), mid HSC signature score (>0 and <0.5), and low HSC signature score (<0) clusters, and cell identities were determined by transcriptional signatures using Celltypist^41^. Cells with a high HSC signature score were enriched for HSCs and cMPPs. Abbreviations of cell types defined in Fig. 2a. **(d)** Stacked bar graph showing *AAVS1* or *MECOM* edited CD34^+^CD45RA^−^CD90^+^ HSCs stratified by expression of HSC signature score as defined in Extended Data Fig. 2c. *MECOM* editing leads to a depletion of cells with high HSC signature score. **(e-g)** UMAP plots of the normalized expression of *CD34* **(e)**, *HLF* **(f)**, and *CRHBP* **(g)** in phenotypic LT-HSCs. The combined expression of these three genes defines the HSC signature in Fig. 2d, e and Extended Data Fig. 2c, d. Source data

Having established a primary human HSC model of *MECOM* haploinsufficiency, we sought to gain insights into the transcriptional circuitry required for human HSC maintenance by single-cell RNA sequencing (scRNA-seq) before complete HSC loss. Three days after *AAVS1* or *MECOM* perturbation, we sorted CD34^+^CD45RA^−^CD90^+^ HSPCs and performed scRNA-seq using the 10x Genomics platform. We used Celltypist^22^ to delineate cellular identity based on lineage-specific signatures and identified 11 cell clusters (Fig. 2a), of which only the earliest HSC cluster was significantly depleted after *MECOM* editing (Fig. 2b,c and Extended Data Fig. 2a). Next we examined cells expressing an HSC molecular signature (*CD34*, *HLF* and *CRHBP*)^23^, which is found in a rare subpopulation representing only 0.6% of 263,828 UCB cells from the Immune Cell Atlas (Extended Data Fig. 2b,c). *MECOM* perturbation led to a significant loss of cells expressing the HSC signature (Fig. 2d,e and Extended Data Fig. 2d). To examine the gene expression changes in this population of transcriptional LT-HSCs, we again edited UCB CD34^+^ HSPCs and sorted for phenotypic CD34^+^CD45RA^−^CD90^+^CD133^+^EPCR^+^ITGA3^+^ LT-HSCs. We found that our sorted phenotypic LT-HSCs are highly enriched for the HSC signature (Fig. 2f and Extended Data Fig. 2e–g). Next, we compared the transcriptomes of 5,935 *MECOM*-edited and 4,291 *AAVS1*-edited phenotypic LT-HSCs. Following our stringent immunophenotypic sorting strategy, *MECOM*-edited LT-HSCs colocalized with *AAVS1*-edited cells (Fig. 2g). This confirmed that our sorting strategy would allow us to directly compare developmentally stage-matched cells before they are completely lost, to uncover transcriptional changes that underlie the profound depletion of LT-HSCs after *MECOM* editing.

As an orthogonal approach to simultaneously profile the precise genomic editing outcome and transcriptional profile of LT-HSCs, we employed genome and transcriptome sequencing (G&T-seq)^24^. *MECOM* heterozygous cells (Fig. 1d) colocalize with *AAVS1*-edited cells, as well as the non-genotyped cells examined with the 10x Genomics method (Fig. 2h). These results reveal a high degree of similarity in the high-dimensional transcriptomic analysis of LT-HSCs following *MECOM* perturbation, as expected given the stringent phenotypic sorting strategy we employed before scRNA-seq analysis. Furthermore, these results suggest that the profound functional consequences of MECOM loss are due to coordinated expression changes in a select group of genes.

### MECOM loss in LT-HSCs elucidates a dysregulated gene network

**Fig. 3:**
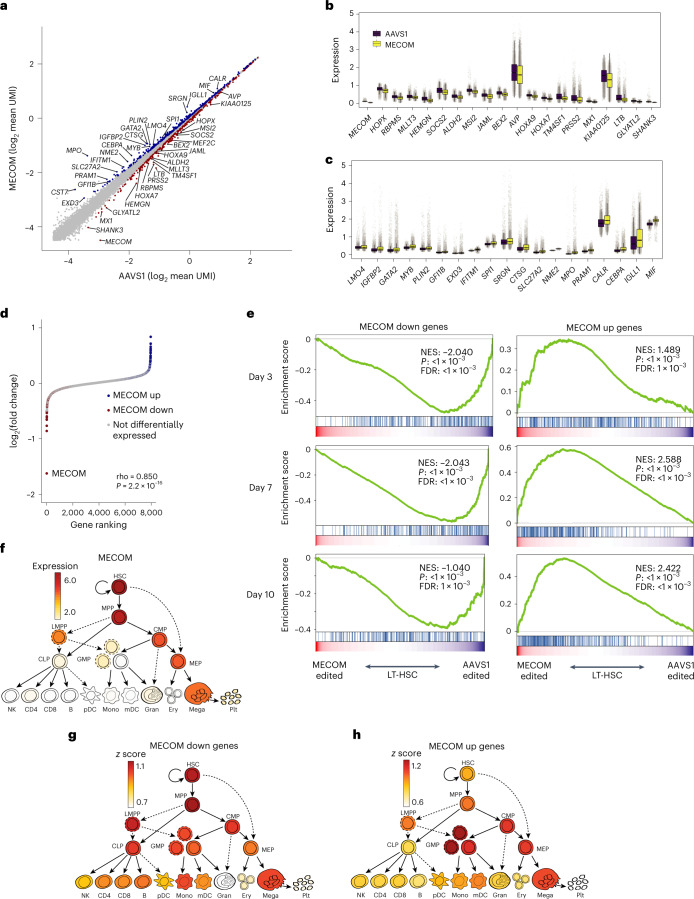
Delineation of a MECOM regulatory network in LT-HSCs. **a**, Scatter-plot of gene expression in LT-HSCs following *AAVS1* or *MECOM* editing. Single-cell expression data for each gene was averaged following imputation and the subset of genes with highest expression is plotted. Differential gene expression was determined using Seurat 4.0 differential expression analysis with the MAST pipeline and is indicated by colored dots, MECOM down genes, red; MECOM up genes, blue. A gene is defined as differentially expressed if log_2_ fold change >0.05 and adjusted *P* < 1 × 10^−20^ as determined by MAST. **b**,**c**, Box plots showing expression of a subset of MECOM down (**b**) and MECOM up (**c**) genes after *MECOM* editing. Gray dots show imputed gene expression in single cells; *n* = 4,291 single cells in the *AAVS1*-edited group and 5,935 cells in the *MECOM*-edited group. The box plot center line, limits and whiskers represent the median, quartiles and interquartile range, respectively. **d**, Pseudobulk analysis of differentially expressed genes. Transcriptomic data from single LT-HSCs that had undergone *AAVS1* or *MECOM* perturbation were integrated to generate pseudobulk gene expression profiles. Expression differences between the *AAVS1* and *MECOM* pseudobulk samples are plotted in rank order and differentially expressed genes from the scRNA-seq analysis are highlighted (MECOM down genes, red; MECOM up genes, blue). Correlation of differential gene expression between pseudobulk and single-cell analyses was calculated using Spearman’s rank correlation and significance was calculated using permutation testing. **e**, GSEA plots showing the depletion of MECOM down genes and the enrichment of *MECOM* up genes in LT-HSCs at three time points in culture after *MECOM* editing. UCB CD34^+^ cells underwent CRISPR editing and were kept in HSC medium with UM171 for the indicated time. The Kolmogorov–Smirnov (K–S) test was used to determine the significance of GSEA. **f**–**h**, Expression of *MECOM* (log_2_ normalized counts per million mapped reads) throughout hematopoietic differentiation reveals robust expression in HSCs (**f**), similar to the enrichment of expression of MECOM down genes (**g**) and the inverse of the expression pattern of MECOM up genes (**h**).

**Extended Data Fig. 3:**
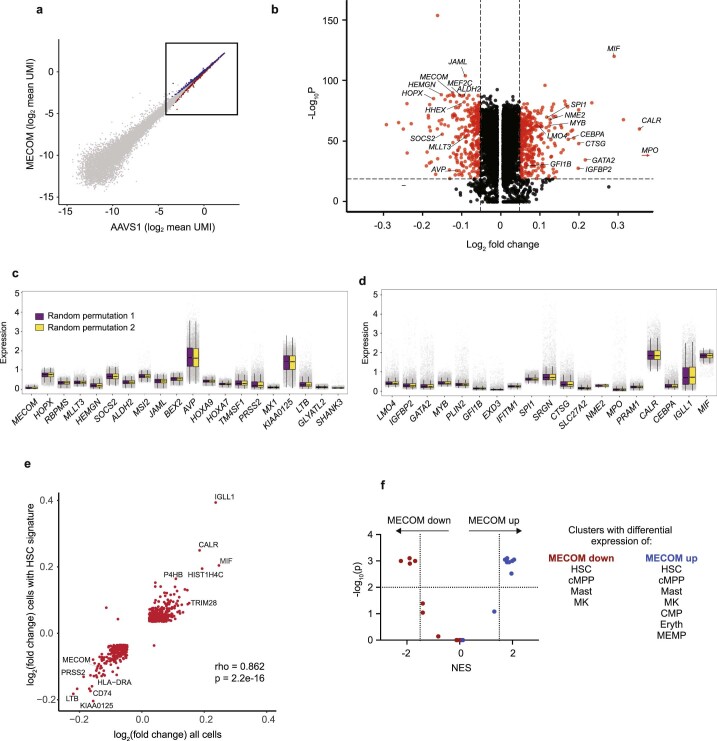
Characterization of the MECOM network in LT-HSCs. **(a)** Scatter plot of gene expression in LT-HSCs following *AAVS1* or *MECOM* editing showing the expression of all genes. The inset box highlights the subset of genes described in Fig. 3a that contains the differentially expressed genes that make up the MECOM regulatory network. (**b)** Volcano plot projection of the data from Fig. 3a displaying the small but significant fold changes in gene expression of MECOM down genes (log_2_ fold change < −0.05) and MECOM up genes (log_2_ fold change > 0.05) with *p*-value <1e-20 as determined by Mann-Whitney U test. Log_2_fold change of MPO expression is out of scale of the axis and is noted by a red arrow. **(c-d)** Box plots showing expression of a subset of MECOM down **(c)** and MECOM up **(d)** genes in a representative random permutation of cohort assignments, demonstrating no difference in gene expression. Gray dots show imputed gene expression in single cells. *n* = 1,000 randomly assigned cells in each group. The box plot center line, limits, and whiskers represent the median, quartiles, and interquartile range, respectively. **(e)** Scatter plot of gene expression in CD34^+^CD45RA^−^CD90^+^CD133^+^EPCR^+^ITGA3^+^ LT-HSCs enriched for the HSC signature compared to bulk LT-HSCs. Expression differences between *MECOM* and *AAVS1*-edited LT-HSCs were calculated and MECOM down and MECOM up genes are plotted. Correlation was calculated using Spearman’s rank correlation test and significance was calculated using permutation testing. **(f)** Scatter plot of enrichment scores of MECOM down and MECOM up gene sets in hematopoietic progenitors. CD34^+^CD45RA^−^CD90^+^ HSCs from Fig. 2b were clustered by cell identities as in Fig. 2a. Differences in gene expression between *AAVS1* and *MECOM* edited samples in each cluster were calculated and used to query for the enrichment of MECOM down (red) or MECOM up (blue) gene sets by GSEA. X-axis plots the Normalized Enrichment Score (NES) and y-axis plots -log_10_(p-value) for each cluster as calculated by Kolmogorov Smirnov (K-S) test. Significant enrichment was defined as NES > 1.5 or < −1.5 and pval <0.01. Source data

To compare individual gene expression in single LT-HSCs following *AAVS1* or *MECOM* editing, we used model-based analysis of single-cell transcriptomes (MAST)^25^ (Fig. 3a and Extended Data Fig. 3a,b). Despite the high-dimensional transcriptional similarity in the LT-HSCs, we detected significant downregulation of a group of 322 genes following *MECOM* editing that we refer to as ‘MECOM down’ genes (Supplementary Table 2), which includes factors with previously described functions in HSC maintenance (Fig. 3a,b). We then used MAST to identify 402 genes that are significantly upregulated after *MECOM* editing, which we refer to as the ‘MECOM up’ gene set (Supplementary Table 2), which includes key factors expressed during hematopoietic differentiation (Fig. 3a,c). To validate these subtle differences, we performed random permutation analysis and did not detect any differentially expressed genes (Extended Data Fig. 3c,d).

To minimize the potential confounding influence of allelic dropout, we performed pseudobulk analysis of gene expression changes following *MECOM* perturbation^26^. We observed that the MECOM down and up gene sets again represented the most differentially expressed genes with larger expression differences compared to the single-cell analysis (Fig. 3d). To validate that the gene expression differences that we observed in the population of immunophenotypic LT-HSCs accurately represented gene expression changes in molecularly defined LT-HSCs, we examined expression of each differentially expressed gene in the subset of cells with robust expression of the HSC signature. There was significant correlation of gene expression changes in this subpopulation of transcriptionally defined LT-HSCs compared to the total population of immunophenotypic LT-HSCs, demonstrating that MECOM network genes were indeed differentially expressed in cells with a stringent molecular HSC signature (Extended Data Fig. 3e). As further validation of this gene signature, we examined differential gene expression in bulk phenotypic LT-HSCs at days 3, 7 and 10 after *MECOM* perturbation and detected significant and consistent changes of the MECOM down and MECOM up gene sets at all time points (Fig. 3e).

Next, we sought to uncover differential gene expression patterns between *AAVS1*- and *MECOM*-edited HSPCs in each of the 11 hematopoietic cell clusters identified in our initial scRNA-seq profiling of CD34^+^CD45RA^−^CD90^+^ cells. The MECOM down genes were significantly depleted from the HSC and cycling multipotent progenitor clusters, but not in other early progenitor populations, including megakaryocyte-erythroid progenitors, megakaryocyte-erythroid-mast cell progenitors and common myeloid progenitors. Early megakaryocytes and mast cell progenitors also had differential expression of MECOM down genes (Extended Data Fig. 3f). Combining these data with the observed cell numbers in each cell cluster after *MECOM* perturbation revealed that only the HSC cluster was depleted (Extended Data Fig. 2a), providing further support for the notion that the MECOM down gene set is crucial for HSC maintenance. Gene set enrichment analysis (GSEA) for the MECOM up genes in each cluster revealed that these genes were significantly enriched in 7 out of the 11 cell clusters (Extended Data Fig. 3f), suggesting that MECOM up genes are expressed in cells undergoing differentiation into multiple lineages. We then evaluated the expression of the MECOM down and up genes during normal hematopoiesis by comparing the enrichment of the gene sets in 20 distinct hematopoietic cell lineages^27^. Similar to *MECOM* itself (Fig. 3f), the MECOM down genes are collectively more highly expressed in HSCs and early progenitors (Fig. 3g). Conversely, the MECOM up genes are turned on during hematopoietic differentiation and are more highly expressed in differentiated cells of various lineages (Fig. 3h). Collectively, these analyses reveal that MECOM loss in LT-HSCs leads to functionally significant transcriptional dysregulation in genes that are fundamental to HSC maintenance and differentiation.

### Increased *MECOM* expression rescues HSC dysregulation

**Fig. 4:**
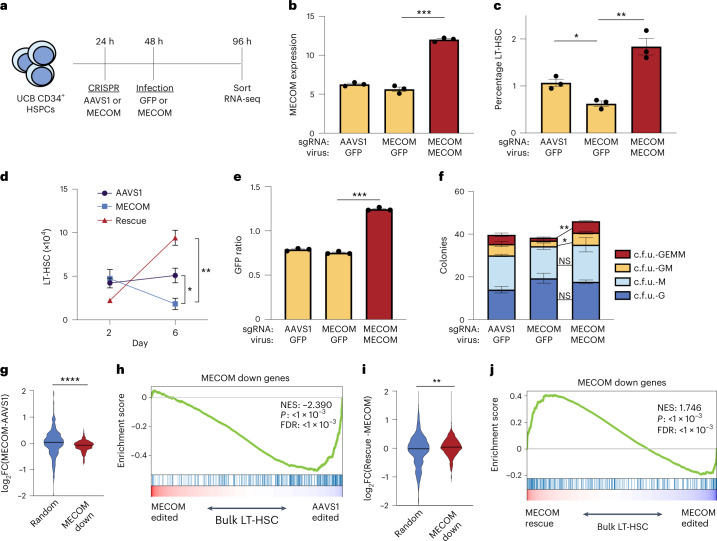
MECOM rescue of functional and transcriptional changes in HSCs. **a**, Experimental outline of *MECOM* editing and rescue. **b**–**d**, Effects of *MECOM* editing and infection with *MECOM* or GFP lentivirus. *MECOM* expression (RPKM) measured by RNA-seq is shown (**b**). Percent of LT-HSC determined by FACS (**c**) and number of LT-HSCs are shown (**d**); *n* = 3 per group. Mean is plotted and error bars show s.e.m. Two-sided Student’s *t*-test was used. *P = 1.1 × 10^−2^, **P = 6.7 × 10^−3^, ***P = 1 × 10^−4^. **e**, GFP ratio following lentiviral infection. GFP ratio is defined as percent of GFP^+^ LT-HSCs divided by the percent GFP^+^ bulk HSPCs. GFP ratio >1 is consistent with enrichment of infected cells in the LT-HSC population; *n* = 3 per group. Mean is plotted and error bars show s.e.m. Two-sided Student’s *t*-test was used. ***P = 1.5 × 10^−4^. **f**, Stacked bar plots of colony-forming assay. Infection with *MECOM* virus leads to restoration of multipotent c.f.u. GEMM and bipotent c.f.u. GM colonies that are lost following *MECOM* editing; *n* = 3 per group. Mean colony number is plotted and error bars show s.e.m. Two-sided Student’s *t*-test was used. *P = 3.3 × 10^−2^, **P = 1.1 × 10^−3^. **g**, Violin plot of differential gene expression in bulk LT-HSCs following *MECOM* perturbation. MECOM down genes are significantly depleted in *MECOM*-edited samples compared to *AAVS1*-edited samples, unlike a set of randomly selected genes. Two-sided Student’s *t*-test was used. **** *P* = 1 × 10^−4^. **h**, GSEA of MECOM down genes after *MECOM* perturbation. MECOM down genes that were identified from scRNA-seq analysis are depleted in *MECOM*-edited LT-HSCs in bulk, compared to *AAVS1*-edited cells. The K–S test was used to determine the significance of GSEA. **i**, Violin plot of differential gene expression in bulk LT-HSCs following *MECOM* perturbation and rescue. MECOM down genes are significantly enriched in *MECOM* rescue samples compared to *MECOM*-edited samples, unlike a set of randomly selected genes. Two-sided Student’s *t*-test was used. **P = 4.7 × 10^−3^. **j**, GSEA of MECOM down genes after *MECOM* perturbation and rescue. MECOM down genes that were identified from the scRNA-seq analysis are enriched in *MECOM* rescued LT-HSCs in bulk, compared to *MECOM*-edited cells. The K–S test was used to determine the significance of GSEA. Source data

**Extended Data Fig. 4:**
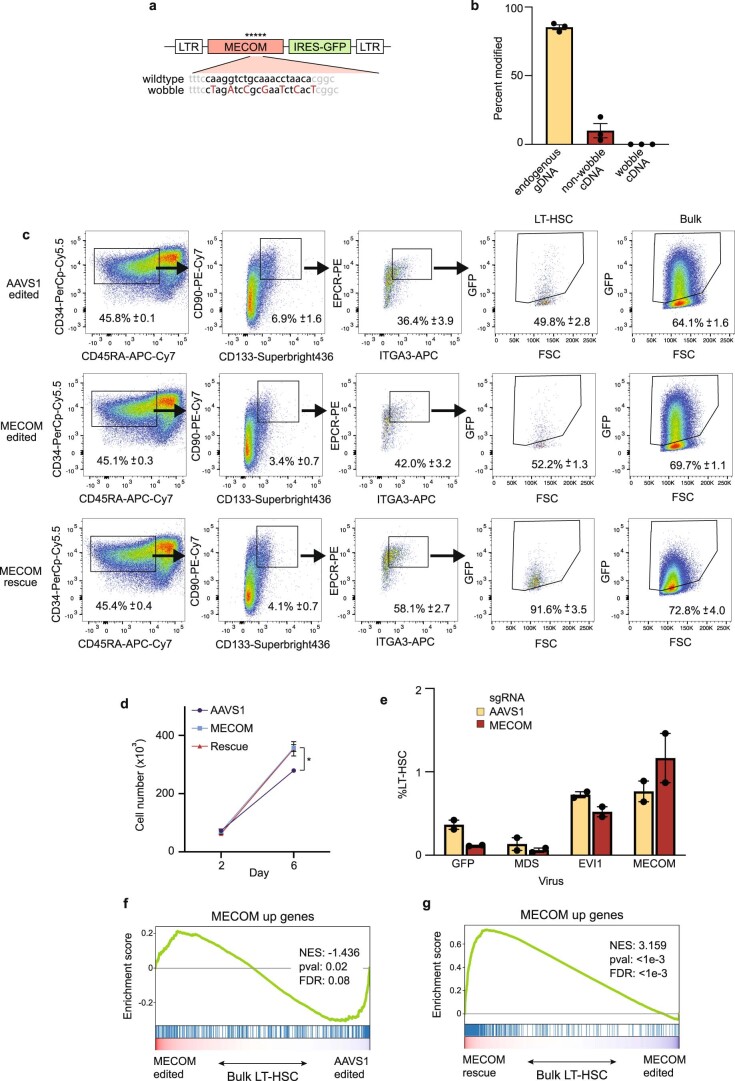
Lentiviral expression of *MECOM* rescues LT-HSCs but does not reverse upregulation of MECOM up genes. **(a)** Schematic of lentiviral vector for increased *MECOM* expression. MECOM sgRNA binding site is shown in bold, and wobble mutations introduced by PCR are indicated. LTR, long terminal repeat; IRES, internal ribosome entry site. **(b)** Edited allele frequency of intended endogenous *MECOM* locus and *MECOM* cDNA after viral integration. Editing and infection were performed as in Fig. 4a. Integrated viral cDNA was amplified using a forward primer in the cDNA sequence and reverse primer in the IRES sequence. *n* = 3 biologically independent samples. Mean is plotted and error bars show s.e.m. **(c)** FACS plots for LT-HSC detection after *MECOM* editing and rescue. Gating strategy as in Fig. 1e. Percentages show the mean (± s.e.m) of three independent experiments. GFP ratio **(**Fig. 4e**)** is defined as the ratio of GFP^+^ cells in LT-HSC population (column 4) to GFP^+^ cells in the bulk population (column 5). **(d)** Cell expansion after *MECOM* editing and rescue. Increased expansion of HSPCs after *MECOM* editing is not reversed by viral *MECOM* expression. AAVS1, edited at *AAVS1*, infected with GFP virus; MECOM, edited at *MECOM*, infected with GFP virus; rescue, edited at *MECOM*, infected with *MECOM* virus, *n* = 3 for each group. Mean is plotted and error bars show s.e.m. Two-sided Student *t*-test used. *P = 3.7e-3. **(e)** Bar graph of the effect of MECOM isoform overexpression on the maintenance of LT-HSCs. HSPCs were edited at *AAVS1* (yellow) or *MECOM* (red) and infected with lentivirus encoding GFP or MECOM isoforms as displayed. The percentage of LT-HSCs was determined by FACS. *n* = 2 biologically independent sample. Mean is plotted and error bars show s.e.m. **(f-g)** GSEA of MECOM up genes after editing and rescue in bulk LT-HSCs. **(f)** MECOM up genes are more highly enriched in AAVS1 samples in bulk in contrast to data from single cell analysis (Fig. 3a). **(g)** MECOM up genes are further increased after MECOM viral infection. The Kolmogorov Smirnov (K-S) test was used to determine the significance of GSEA. Source data

To confirm that the functional and transcriptional impacts on LT-HSCs are due specifically to reduced MECOM levels, we sought to rescue the phenotype by lentiviral *MECOM* expression in HSCs after CRISPR editing (Fig. 4a). To avoid unintended CRISPR disruption of the virally encoded *MECOM* complementary DNA, we introduced wobble mutations in the single guide RNA (sgRNA) binding site in the cDNA (Extended Data Fig. 4a,b). Infection of *MECOM*-edited HSPCs with *MECOM* virus led to supraphysiologic levels of *MECOM* expression (Fig. 4b), which was sufficient to rescue the LT-HSC loss observed after *MECOM* editing (Fig. 4c,d and Extended Data Fig. 4c,d). Expression of the shorter *MECOM* isoform *EVI1* resulted in a higher percentage of LT-HSCs on day 6, but this increase was blunted by endogenous *MECOM* editing. Expression of the *MDS* isoform did not result in rescue of LT-HSCs (Extended Data Fig. 4e). Green fluorescent protein (GFP) is coexpressed with *MECOM* and we observed a significantly higher ratio of GFP expression in LT-HSCs compared to the bulk population (Fig. 4e), confirming that increased *MECOM* expression favored LT-HSC preservation. Increased *MECOM* expression also rescued the loss of multipotent and bipotent progenitor colonies after *MECOM* editing (Fig. 4f). Together, these data reveal that restoration of the full-length *MECOM* isoform is sufficient to overcome the functional loss of LT-HSCs caused by endogenous *MECOM* perturbation.

Next, we performed RNA-seq of phenotypic LT-HSCs after *MECOM* editing and rescue. After *MECOM* perturbation alone, we observed significantly lower expression of the MECOM down gene set compared to a subset of randomly selected genes (Fig. 4g). Similarly, GSEA revealed significant depletion of the MECOM down genes (Fig. 4h). Following rescue by increasing *MECOM* expression, the MECOM down genes were significantly upregulated (Fig. 4i,j and Supplementary Table 3). While increasing *MECOM* expression can rescue the impact of *MECOM* perturbation in short-term in vitro contexts, due to the risk of leukemic transformation driven by constitutive *MECOM* overexpression^12^, it is challenging to assess this rescue of HSC function in vivo.

We did not observe upregulation or subsequent rescue of the MECOM up genes in bulk following *MECOM* perturbation and overexpression (Extended Data Fig. 4g,h). The MECOM up gene set contains factors important for hematopoietic differentiation. Lentiviral infection may subtly alter this process. Alternatively, the supraphysiologic expression that we obtained may not allow effective regulation of the MECOM up genes. Regardless, these data collectively show that the loss of LT-HSCs after *MECOM* editing can be rescued with increased *MECOM* expression and is accompanied by restoration of the MECOM down gene set.

### Defining the HSC *cis*-regulatory network mediated by MECOM

**Fig. 5:**
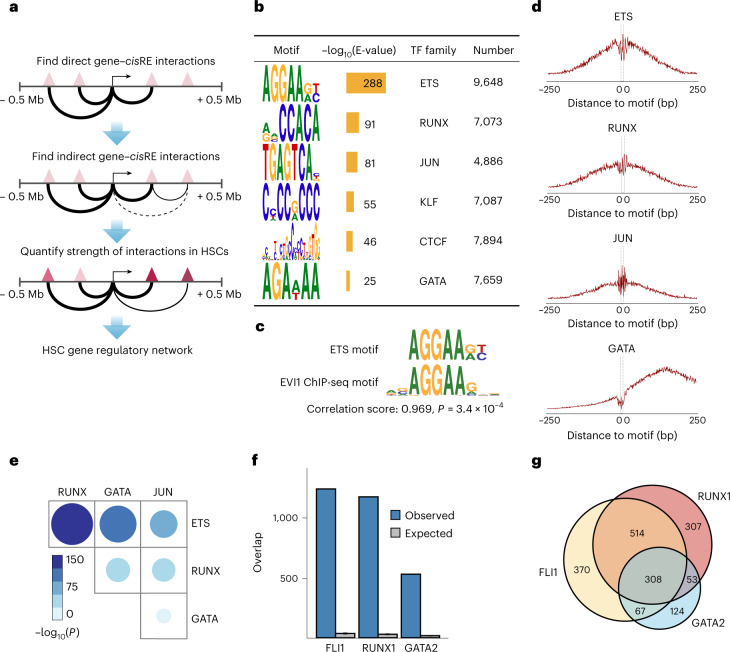
Defining the HSC *cis*-regulatory network coordinated by MECOM. **a**, Schematic of the HemeMap method used to define an HSC-specific regulatory network. **b**, Significantly enriched conserved motifs associated with *cis*REs of MECOM network genes in the HSC-specific regulatory network and the number of instances of each motif are shown. Motif discovery and significance testing were performed using MEME. **c**, Motif similarity between the ETS motif and a previously identified EVI1 motif from ChIP-seq^13^. Similarity was determined by the Pearson correlation coefficient of the position frequency matrix in a comparison of the two motifs and significance was determined using permutation test. **d**, Footprinting analysis of ETS, RUNX, JUN and GATA within the *cis*REs in the MECOM regulation network. The plots show Tn5 enzyme cleavage probability of each base flanking (±250 bp) and within TF motifs in HSCs. **e**, Analysis of TF footprint co-occurrence in the MECOM network. The frequency of occurrence of each footprint in MECOM network *cis*REs was computed and the *P* value of co-occurrence for each TF pair was determined by a two-sided hypergeometric test. The color and size of dots are proportional to statistical significance. **f**, Specific TF occupancy of *cis*REs in the MECOM network in CD34^+^ HSPCs. The number of *cis*REs associated with the MECOM network that overlap with ChIP-seq peaks for FLI1, RUNX1 and GATA2 were determined. For each TF, the expected distribution of overlapping *cis*REs was generated by 1,000 permutations of an equal number of TF peaks across the genome. Mean is plotted and error bars show s.d. **g**, Overlap of TF occupancy in MECOM network *cis*REs. The number of *cis*REs that contain ChIP-seq peaks for FLI1 (yellow), RUNX1 (red), GATA2 (blue) or combinations of TFs are indicated.

**Extended Data Fig. 5:**
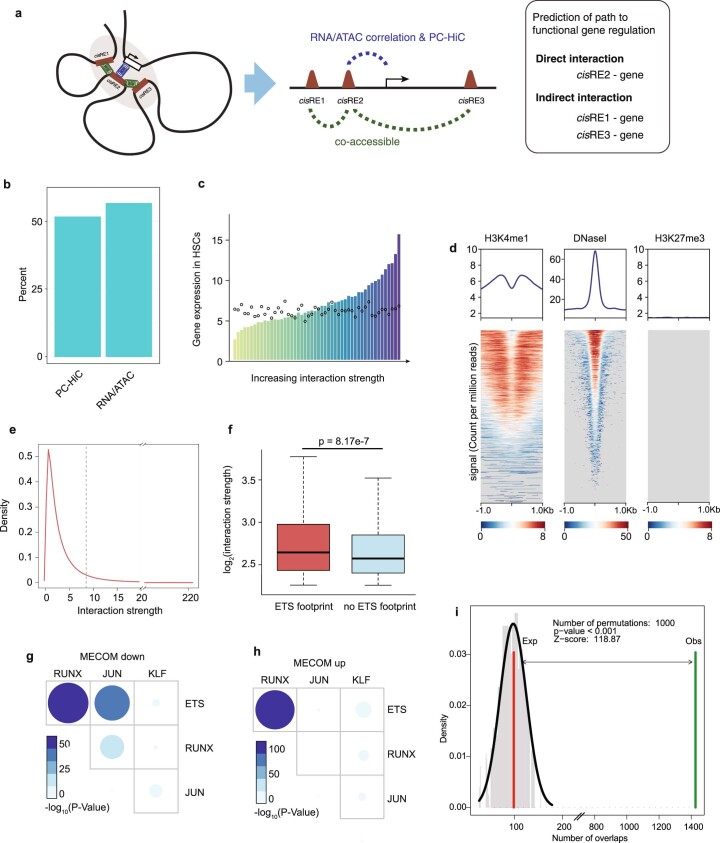
Establishment of a *cis*-regulatory network in HSCs. **(a)** Schematic view demonstrating different types of functional interactions between *cis*-regulatory elements and genes. HemeMap predicts these interactions by integration of multiomics data including RNAseq, ATACseq and promoter capture-HiC (PC-HiC) data across 16 or 18 hematopoietic cell types. **(b)** Bar graph showing the overlap between genomic interactions nominated by HemeMap and experimentally defined interactions. More than half of the direct interactions nominated by PC-HiC and RNA^−^ATAC correlations were supported by evidence from Hi-C interactions in HSPCs. **(c)** Correlation of *cis*RE-gene interaction strength with gene expression in HSCs. HemeMap scores were calculated for each *cis*REs-gene interaction and HemeMap interactions were arranged by increasing scores and grouped evenly into 50 bins. Median gene expression in each bin is depicted (bars). The median expression of a randomly sampled equal-sized gene set is shown (dots). **(d)** Validation of *cis*REs associated with MECOM network genes. H3K4 methylation, DNase hypersensitivity and H3K27 trimethylation signals from HSPCs^52^ at MECOM network *cis*REs reveals an active transcriptional pattern consistent with enhancer elements. **(e)** Distribution of HemeMap scores in HSCs. To construct the HSC-specific regulatory network, significant interaction scores >8.91 were included. Significance threshold was determined by Chi-square distribution. **(f)** Comparison of interaction strengths. *cis*REs containing ETS footprint (*n* = 711) were significantly associated with stronger HemeMap scores than those without (*n* = 6,371). P-values as shown were calculated using a two-sided Wilcoxson signed-rank test. The box plot center line, limits, and whiskers represent the median, quartiles and 1.5x interquartile range, respectively. **(g-h)** Analysis of TF footprint co-occurrence in the *cis*REs associated with MECOM down genes **(g)** and MECOM up genes **(h)**, respectively. The frequency of occurrence and *P* values were calculated using a two-sided hypergeometric test. The color and size of dots are proportional to statistical significance. **(i)** Experimentally defined EVI1 ChIP-seq peaks^26^ were compared to HemeMap predicted *cis*REs of MECOM network genes and show significant overlap with *cis*REs that contain ETS footprints. P-value was determined by permutation testing.

We next sought to define the *cis*-regulatory elements (*cis*REs) that control expression of the MECOM network, which underlies HSC self-renewal. To do so, we developed HemeMap, a computational framework to identify putative *cis*REs and cell-type-specific *cis*RE-gene interactions by integrating multiomic data from 18 hematopoietic cell populations (Fig. 5a and Extended Data Fig. 5a,b)^28–32^. We calculated HemeMap scores based on chromatin accessibility for each *cis*RE-gene interaction in HSCs and found that the scores were correlated with gene expression (Extended Data Fig. 5c). There was significant overlap of the predicted enhancer–gene pairings from HemeMap with chromatin looping data in hematopoietic progenitors^29^ and predicted regulatory elements in HSPCs^33^. Our *cis*REs had a strong H3K4me1 signal and DNase hypersensitivity without an H3K27me3 signal, consistent with their likely identities as enhancer elements (Extended Data Fig. 5d). All of the interactions with a significant HemeMap score in HSCs were selected to construct an HSC-specific regulatory network (Extended Data Fig. 5e).

To identify cooperating transcription factors (TFs) driving expression of the MECOM network genes in HSCs, we performed unbiased motif discovery within the MECOM network *cis*REs and found six significantly enriched motifs: ETS, RUNX, JUN, KLF, CTCF and GATA (Fig. 5b). The ETS family motif (AGGAAGT) was most highly enriched and can be bound by several hematopoietic TFs, including FLI1, ERG, ETV2 and ETV6 (ref. ^34^). Additionally, the experimentally determined binding motif of EVI1 in AML^13^, is a near perfect mimic of our nominated ETS motif, suggesting that many of these *cis*REs may be directly occupied by MECOM (Fig. 5c). Notably, HemeMap scores were significantly higher in *cis*REs with ETS motifs compared to those without (Extended Data Fig. 5f).

Next, we performed digital genomic footprinting analyses to predict TF occupancy in HSCs (Supplementary Tables 4 and 5 and Fig. 5d). We observed a significant co-occurrence of footprints across TF pairs, with a particular enrichment of overlap between ETS with RUNX, JUN and GATA footprints, suggesting cooperativity between these TFs (Fig. 5e and Extended Data Fig. 5g,h). We evaluated specific TF binding to the MECOM network *cis*REs by integrating TF ChIP-seq data from human HSPCs^35^. Consistent with the footprinting analysis, we found highly enriched TF occupancy of the ETS family member FLI1, as well as RUNX1 and GATA2 in HSPCs (Fig. 5f). These ChIP-seq data are derived from bulk CD34^+^ HSPCs, so while they provide a general indication of TF binding in HSPCs, there may be important differences in TF binding in LT-HSCs. As further evidence of TF cooperativity, we found that FLI1, RUNX1 and GATA2 have significant co-occupancy at the MECOM-regulated gene *cis*REs in HSPCs (Fig. 5g). Additionally, we examined EVI1 binding data from overexpression studies^14^ and found significant overlap with *cis*REs that contain ETS footprints (Extended Data Fig. 5i). These analyses from heterogenous populations of hematopoietic progenitors provide support for our model of cooperativity between MECOM and other hematopoietic TFs (these datasets are summarized in Supplementary Table 6).

### Dynamic CTCF binding represses MECOM down genes

In addition to the enrichment of HSC TF motifs, the MECOM network *cis*REs showed CTCF motif enrichment. CTCF is a regulator of three-dimensional genome organization and acts by anchoring cohesin-based chromatin loops to insulate genomic regions of self-interaction^36^. Recently, CTCF has been implicated in regulating HSC differentiation by altering looping to silence key stemness genes^37^, while also cooperating with lineage-specific TFs during hematopoietic differentiation^38^. Therefore, we hypothesized that CTCF plays a role in mediating the differential expression of MECOM down genes following loss of *MECOM*.

**Fig. 6:**
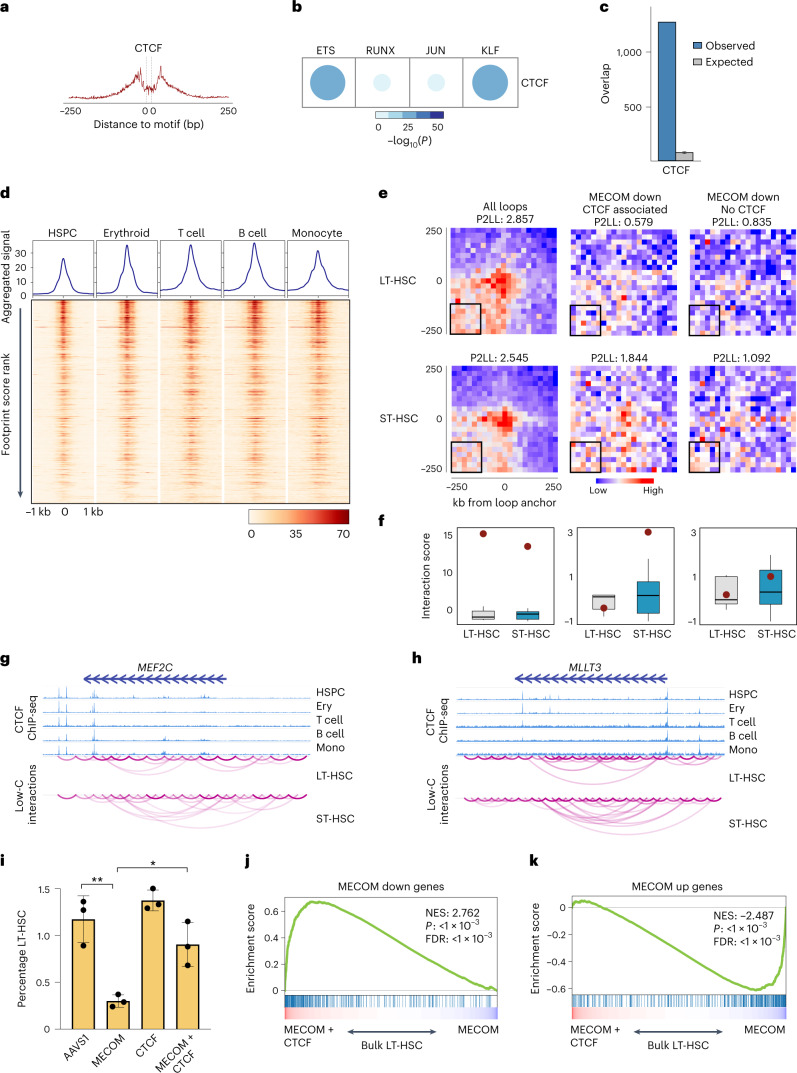
Dynamic CTCF binding facilitates repression of MECOM down genes as HSCs undergo differentiation. **a**, Footprinting analysis of CTCF within the *cis*REs in the *MECOM* gene network. The plot shows Tn5 enzyme cleavage probability for each base flanking (±250 bp) and within the CTCF motif. **b**, Analysis of TF footprint co-occurrence of CTCF and other TFs in *cis*REs associated with MECOM down genes. The frequency of occurrence and *P* values were calculated using a two-sided hypergeometric test. The color and size of dots are proportional to statistical significance. **c**, CTCF occupancy of *cis*REs in MECOM down genes in CD34^+^ HSPCs. The number of *cis*REs associated with MECOM down genes that overlap with CTCF ChIP-seq peaks was determined and plotted as in Fig. 5f. The expected distribution of overlapping *cis*REs was generated by 1,000 permutations of an equal number of TF peaks across the genome. Mean is plotted and error bars show s.d. **d**, CTCF binding to MECOM down *cis*REs in hematopoietic lineages. Heat maps (bottom) show the CTCF ChIP-seq signals that overlap CTCF footprints in MECOM down *cis*REs in HSPCs, erythroid cells, T cells, B cells and monocytes. Each row represents a footprint ±1 kb of flanking regions and the rows are sorted by the posterior probability of footprint occupancy from high to low. The enrichment of CTCF binding to *cis*REs was calculated and displayed in the line graph (top). **e**, Aggregate peak analysis for the enrichment of chromatin loops in LT-HSCs (top) and ST-HSCs (bottom) using Low-C data. Chromatin loop interactions were determined for all chromatin loops derived from Hi-C data in hematopoiesis (left), the subset of CTCF-associated loops of MECOM down genes (center) and the subset of non-CTCF-associated loops of MECOM down genes (right). Aggregate signals over 500 kb centered on loop anchors with 25-kb resolution were calculated and are shown. The peak to lower left ratio (P2LL) enrichment score was calculated by comparing the peak signal to the mean signal of bins highlighted in black box in the heat map and is shown in the title of each plot. **f**, Box plots showing the standard normalized distribution of interaction scores for the lower left corner highlighted in the heat map in **e**. Red dots indicate the peak value. The columns are as described in **e**. Two-sided Student’s *t*-test was used to compare box plots which revealed no significant differences in background signal. For each box, *n* = 36 interactions and the box plot center line, limits and whiskers represent the median, quartiles and 1.5× interquartile range, respectively. **g**,**h**, Genome browser views of CTCF occupancy and chromatin interaction at *MEF2C* (**g**) and *MLLT3* (**h**) gene loci in LT-HSCs and ST-HSCs. **i**, Bar graphs of LT-HSC rescue by dual *MECOM* and *CTCF* perturbation. Human HSPCs underwent CRISPR editing with the sgRNA guides depicted on the *x* axis. Percent of LT-HSCs was determined by FACS on day 6; *n* = 3 per group. Mean is plotted and error bars show s.e.m. Two-sided Student’s *t*-test was used. *P = 1.3 × 10^−2^, **P = 4.2 × 10^−3^. **j**,**k**, GSEA of MECOM down genes (**j**) and MECOM up genes (**k**) after dual *MECOM* and *CTCF* perturbation compared to *MECOM* perturbation alone. Bulk RNA-seq was performed in biological triplicate on day 5 after CRISPR perturbation. MECOM down genes are enriched and MECOM up genes are depleted following concurrent *CTCF* editing. The K–S test was used to determine the significance of GSEA. Source data

**Extended Data Fig. 6:**
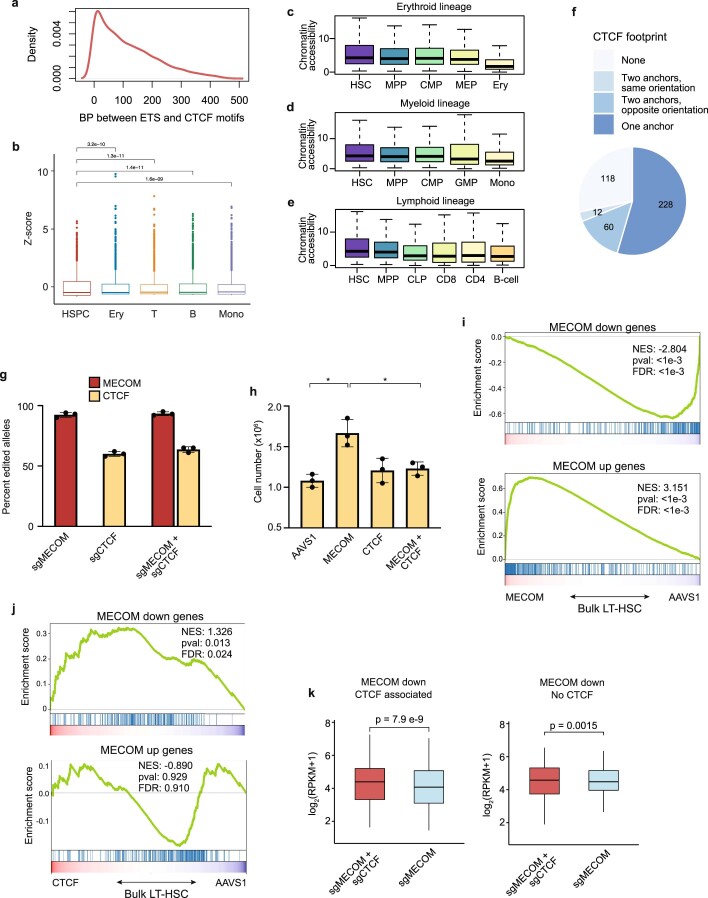
CTCF-mediated looping of MECOM down genes in HSCs. **(a)** Density plot showing the distribution of distance between ETS motifs and CTCF motifs in *cis*REs of MECOM network genes. Average distance is 36 base pairs (BP). **(b)** Box plots depicting the quantitative difference of CTCF ChIP-seq signals between CD34^+^ HSPCs and lineage-committed cells from Fig. 6d. The normalized CTCF ChIP-seq signals of 50 bp regions centered on CTCF footprints (*n* = 6,185) were calculated and compared. The significance was determined using a two-sided Wilcoxson signed-rank test and p-values for each comparison are displayed. The box plot center line, limits, and whiskers represent the median, quartiles, and interquartile range, respectively. **(c-e)** Box plots displaying the chromatin accessibility of CTCF-associated c*is*REs during hematopoietic differentiation. MECOM down *cis*REs that contain a CTCF footprint (*n* = 6,185) are associated with progressively less chromatin accessibility during differentiation along the **(c)** erythroid, **(d)** myeloid, and **(e)** lymphoid lineages. The box plot center line, limits, and whiskers represent the median, quartiles, and interquartile range, respectively. **(f)** Chromatin interactions of MECOM down genes based on the presence and orientation of CTCF footprint. 448 chromatin interactions involving MECOM down genes were identified and were categorized as: (1) no CTCF footprint detected at either anchor (2) CTCF present both anchors in same orientation (3) CTCF present both anchors in opposite orientation (4) CTCF present at only one anchor. **(g)** Bar graphs of CRISPR editing frequencies in human HSPCs. Cells that underwent dual CRISPR perturbation of *MECOM* and *CTCF* had editing similar frequencies compared to single-edited cells. *n* = 3 per group. Mean is plotted and error bars show s.e.m. **(h)** Bar graphs of total cell number following CRISPR editing. Increased expansion of HSPCs following MECOM perturbation was seen as in Extended Data Fig. 1i and was rescued by dual *MECOM* and *CTCF* perturbation. *n* = 3 per group. Mean is plotted and error bars show s.e.m. Two-sided Student *t*-test used.* *P* = 5e-2. **(i)** GSEA of MECOM down genes and MECOM up genes in bulk LT-HSCs after *MECOM* perturbation compared to *AAVS1* perturbation. MECOM down genes are depleted and MECOM up genes are enriched following *MECOM* editing. The Kolmogorov Smirnov (K-S) test was used to determine the significance of GSEA. **(j)** GSEA of MECOM down genes and MECOM up genes in bulk LT-HSCs after *CTCF* perturbation compared to *AAVS1* perturbation. MECOM down genes are upregulated after CTCF editing alone, but there is no enrichment of MECOM up genes. The Kolmogorov Smirnov (K-S) test was used to determine the significance of GSEA. (**k**) Expression of MECOM down genes that are associated with at least two CTCF footprints (*n* = 80, left) and those not associated with CTCF footprints (*n* = 29, right), following either *MECOM* perturbation alone or dual *MECOM* and *CTCF* perturbation. P-values as shown were calculated using a two-sided Wilcoxson signed-rank test. The box plot center line, limits, and whiskers represent the median, quartiles, and interquartile range, respectively. Source data

We uncovered CTCF footprints in bulk CD34^+^ HSPCs (Fig. 6a) and significant co-occurrence of CTCF with ETS, RUNX, JUN and KLF footprints in the *cis*REs of MECOM down genes (Fig. 6b). On average, the distance between ETS and CTCF footprints in our *cis*REs was 36 base pairs (Extended Data Fig. 6a). We observed significant CTCF binding to the nominated *cis*REs (Fig. 6c). We found CTCF occupancy of nominated footprints was highly conserved across erythroid cells, T cells, B cells and monocytes (Fig. 6d and Extended Data Fig. 6b). In HSPCs, CTCF binding was measured in bulk CD34^+^ cells, which contain LT-HSCs and numerous other progenitors. Despite the heterogeneity of the HSPC compartment, terminally differentiated cells showed significantly stronger CTCF signals compared to the CD34^+^ HSPCs and chromatin accessibility at those loci decreased during hematopoietic differentiation (Extended Data Fig. 6c–e). Although these analyses do not allow for a sensitive description of CTCF binding throughout the many intermediate stages of hematopoietic differentiation, they reveal increased binding of CTCF to the *cis*REs of MECOM down genes in differentiated cells in comparison with the heterogenous population of CD34^+^ HSPCs.

To gain mechanistic insights into the role of CTCF in the MECOM-driven regulation of HSC quiescence, we analyzed an overall set of 7,358 chromatin loops from studies of HSCs^37^, as well as a subset of loops whose anchors colocalized with MECOM network *cis*REs. These loops were elucidated in the OCI-AML2 cell line, which was previously used to extrapolate differential looping as LT-HSCs exit quiescence^37^. In total, 448 chromatin interactions were identified for MECOM down genes and the loop anchors showed a strong enrichment of CTCF footprints (Extended Data Fig. 6f). Next, we performed aggregate peak analysis to compare the genomic organization of the MECOM down genes upon exit from quiescence by integrating Low-C chromatin interaction data from phenotypic LT-HSCs and short-term (ST)-HSCs. Using all 7,358 common chromatin loops, there was significant enrichment of chromatin interaction apices in both LT-HSCs and ST-HSCs, as previously observed^37^, but there was no significant difference between the populations. Analysis of the chromatin loops of CTCF footprint-containing *cis*REs associated with MECOM down genes revealed significantly stronger chromatin interactions in ST-HSCs compared to LT-HSCs. There was no chromatin interaction difference in MECOM down genes that lacked association with a CTCF footprint-containing *cis*RE (Fig. 6e,f). These observations are consistent with the concept that CTCF activity at the *cis*REs of MECOM down genes induces tighter chromatin looping and restricts gene expression, promoting differentiation of HSCs, as exemplified by the increased chromatin looping at *MLLT3* and *MEF2C* concordant with their silencing as LT-HSCs differentiate (Fig. 6g,h).

To validate their functional interaction, we performed simultaneous *MECOM* and *CTCF* perturbation in primary human HSPCs (Extended Data Fig. 6g) and observed that concurrent *CTCF* perturbation was sufficient to rescue the loss of LT-HSCs (Fig. 6i) and prevent the increased expansion of HSPCs caused by *MECOM* perturbation (Extended Data Fig. 6h). GSEA revealed significant depletion of MECOM down genes and significant upregulation of MECOM up genes following *MECOM* compared to *AAVS1* editing, corroborating our observations from single cells (Extended Data Fig. 6i). When compared to the *AAVS1* sample, *CTCF* editing alone resulted in significant enrichment of the MECOM down gene set, but no significant changes in the MECOM up genes (Extended Data Fig. 6j). Dual editing of *MECOM* and *CTCF* resulted in significant upregulation of MECOM down genes (Fig. 6j) and significant depletion of MECOM up genes (Fig. 6k). Upon dual perturbation, there was significantly greater rescue of MECOM down genes that are associated with *cis*REs containing CTCF binding motifs compared to those without CTCF motifs (Extended Data Fig. 6k). These data demonstrate that MECOM plays a key role in activating the expression of genes critical for HSC maintenance, which are then subject to genomic reorganization by CTCF upon differentiation.

### The MECOM gene network is hijacked in high-risk AMLs

**Fig. 7:**
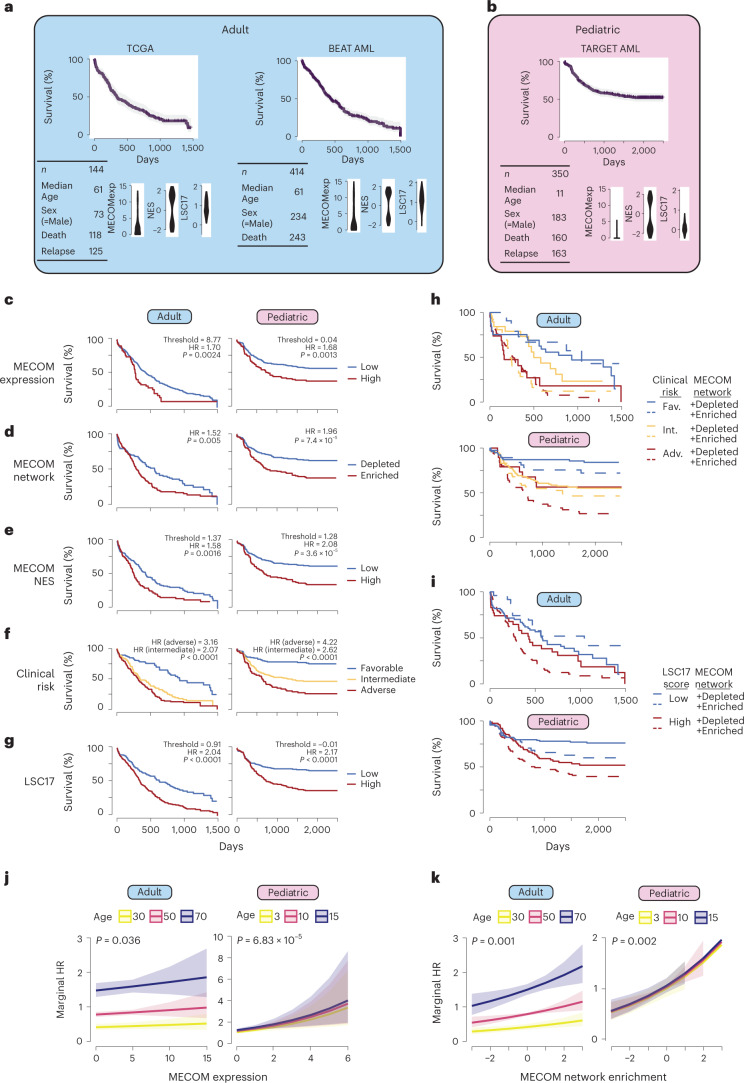
The MECOM down gene network is hijacked in high-risk adult and pediatric AML. **a**,**b**, Descriptive statistics for included clinical cohorts. After correcting for study, TCGA and BEAT data were integrated into an adult cohort (**a**). All of the pediatric data came from the TARGET database (**b**). Distribution of *MECOM* expression, MECOM NES and LSC17 score are displayed for each clinical dataset. **c**–**g**, Kaplan–Meier (KM) overall survival curves for adult and pediatric AML cohorts stratified by *MECOM* expression (**c**), MECOM network enrichment (**d**), MECOM NES (**e**), clinical risk group (**f**) and LSC17 (**g**). For continuous variables in **c**,**e**,**g** optimal threshold was determined by maximizing sensitivity and specificity on mortality (Youden’s *J* statistic). HRs were computed from univariate Cox proportional hazard models. *P* values representing the result of Mantel–Cox log-rank testing are displayed. Test for trend was performed for clinical risk group stratification (more than two groups). **h**,**i**, KM overall survival curves stratified by current prognostic tools and MECOM down network status. MECOM network enrichment was significantly associated with mortality independent of clinical risk group in adult (*P* = 0.005) and pediatric (*P* = 0.008) AML (**h**) and independent of LSC17 score in adult (*P* = 0.01) and pediatric (*P* = 0.01) AML (**i**). **j**,**k**, Marginal hazard of death associated with increasing *MECOM* expression (**j**) and MECOM network enrichment score (**k**), stratified by age. 95% confidence interval of death is shown in the shaded regions. *P* values representing the significance of *MECOM* expression and MECOM network enrichment on survival were calculated using two-sided multivariable Cox proportional hazards modeling, adjusted for age and sex.

**Extended Data Fig. 7:**
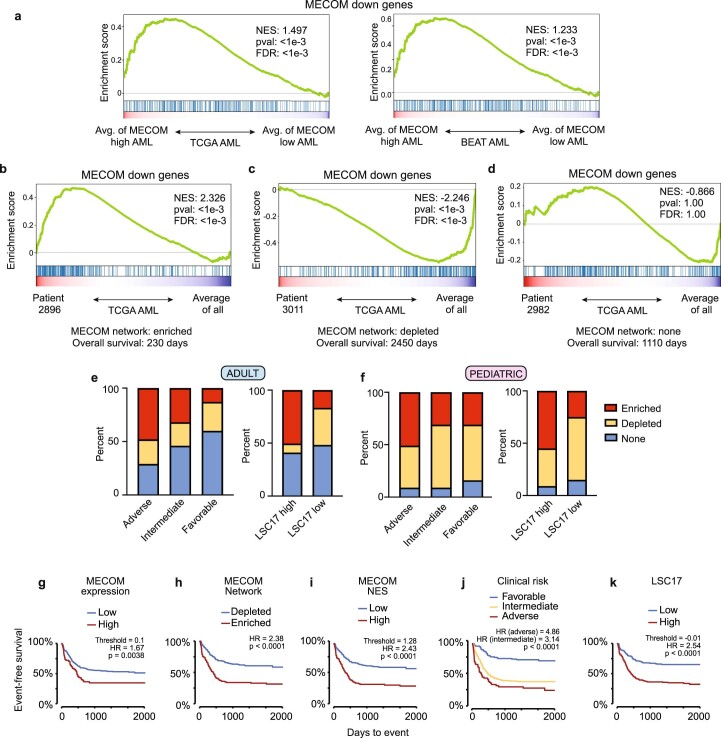
MECOM down gene network enrichment is independently associated with overall and event-free survival. **(a)** GSEA of MECOM down genes in primary AML patient samples from TCGA (left) and BEAT AML (right) stratified by MECOM expression. Individual gene expression was averaged from TCGA or BEAT AML samples with MECOM expression of log_2_(RPKM) > 4 and compared to the average of samples with MECOM expression log_2_(RPKM) < 4. The Kolmogorov Smirnov (K-S) test was used to determine the significance of GSEA. **(b-d)** GSEA of MECOM down genes in primary AML patient samples from TCGA. For each patient sample, expression of every gene was compared to its average expression from all TCGA patient samples, and GSEA was performed to assess for enrichment of MECOM down genes. Representative plots of three individual patients are shown. **(b)** Patient 2896 had enrichment of MECOM down genes and an overall survival of 230 days. **(c)** Patient 3011 had depletion of MECOM down genes and an overall survival of 2450 days. **(d)** Patient 2982 had no significant enrichment or depletion of MECOM down genes and an overall survival of 1110 days. The Kolmogorov Smirnov (K-S) test was used to determine the significance of GSEA. **(e-f)** Stacked bar graph showing proportion of patients with MECOM network enrichment or depletion following stratification by clinical risk group or LSC17 score in adult **(e)** or pediatric AML **(f)**. **(g-k)** KM event-free survival curves for the pediatric AML cohort stratified by **(g)** MECOM expression, **(h)** MECOM network enrichment, **(i)** MECOM NES, **(j)** clinical risk group, and **(k)** LSC17. For continuous variables in **(g)**, **(i)**, and **(k)** the optimal threshold was determined by maximizing sensitivity and specificity on mortality (Youden’s J statistic). Hazard Ratios (HR) were computed from univariate cox-proportional hazard models. P values representing the result of Mantel-Cox log-rank testing are displayed. Test for trend was performed for clinical risk group stratification (>2 groups). Source data

Having elucidated a fundamental transcriptional regulatory network necessary for HSC maintenance, we wondered to what extent this network may be relevant to leukemia. First, we combined 165 primary adult AML samples from The Cancer Genome Atlas (TCGA)^39^ with 430 adult samples from the BEAT AML dataset^40^ into an adult AML cohort (Fig. 7a). We found significant enrichment of the MECOM down gene set in clinical samples with high *MECOM* expression levels (Extended Data Fig. 7a). We analyzed this adult AML cohort in parallel with 440 pediatric AML samples from the TARGET AML dataset^41^ (Fig. 7b). Using optimal thresholding to stratify patients by *MECOM* expression, we observed a survival disadvantage in both adult and pediatric AML (Fig. 7c), consistent with previous reports^42,43^.

Given the importance of the MECOM down gene network in HSC maintenance, we sought to determine whether expression of this network was associated with survival in AML. Using GSEA, we determined whether individual patient AML samples had enrichment or depletion of the MECOM down gene set (Extended Data Fig. 7b–d). Enrichment of the MECOM down gene set was associated with worse survival in both the adult (hazard ratio (HR) 1.52 (95% CI 1.13–2.04), *P* = 0.005) and pediatric AML cohorts (HR 1.96 (95% CI 1.38–2.69), *P* *=* 7.4 × 10^−5^; Fig. 7d).

We then generated a rank order list based on the normalized enrichment score (NES) for each sample to allow for further stratification based on the degree of network enrichment. We used optimal thresholding to stratify patients based on NES and found significantly worse overall survival in patients with high MECOM NES compared to patients with low NES in both adult (HR 1.58 (95% CI 1.18–2.11), *P* = 0.0016) and pediatric (HR 2.08 (95% CI 1.49–2.89), *P* *=* 3.6 × 10^−5^) patients (Fig. 7e).

Stratification based on clinical risk group or LSC17 score^44^ had significant associations with survival (Fig. 7f,g) and we sought to determine whether MECOM network enrichment identified the same subgroup of high-risk patients. We observed that 48% of adult AML and 51% of pediatric AML with adverse clinical risk features also had MECOM network enrichment. Similarly, we found that 51% of adult AML and 55% of pediatric AML with high LSC17 scores had MECOM network enrichment (Extended Data Fig. 7e,f). Thus, MECOM network enrichment identifies a largely unique subset of patients compared to currently available risk stratification tools.

Next, we investigated whether the addition of MECOM network enrichment to the clinical risk group or LSC17 score resulted in improved risk stratification. In the adult AML cohort, MECOM down gene set enrichment was independently associated with mortality particularly in patients with intermediate risk AML (*P* = 0.005) (Fig. 7h) and high LSC17 score (*P* = 0.01) (Fig. 7i). The contribution of MECOM network enrichment to clinical risk grouping was even more striking in the pediatric AML cohort in which MECOM network enrichment was significantly associated with mortality independent of clinical risk group (*P* = 0.008) (Fig. 7h) and, separately, independent of LSC17 score (*P* = 0.01) (Fig. 7i). These results reveal that stratification of primary AML patient samples by MECOM down gene enrichment can be integrated with currently available prognostic tools to improve risk stratification for overall survival in both adult and pediatric AML. Additionally, MECOM down network enrichment was significantly associated with lower event-free survival, independent of clinical risk group and LSC17 score in pediatric AML (*P* = 1.72 × 10^−6^ and *P* = 5.62 × 10^−5^, respectively) (Extended Data Fig. 7g,k).

Finally, we calculated marginal HRs to evaluate the degree of *MECOM* expression or MECOM network NES with overall survival. We observed a modest effect of incremental increases of *MECOM* expression on the marginal HR of survival (Fig. 7j) and a much more significant effect of incremental increases in MECOM NES (Fig. 7k). Together, these data reveal that the MECOM down network is highly enriched in a subset of adult and pediatric AMLs with poor prognosis and can be integrated with currently available prognostic tools to improve risk stratification for patients with AML.

### Validation of MECOM addiction in a subset of high-risk AMLs

**Fig. 8:**
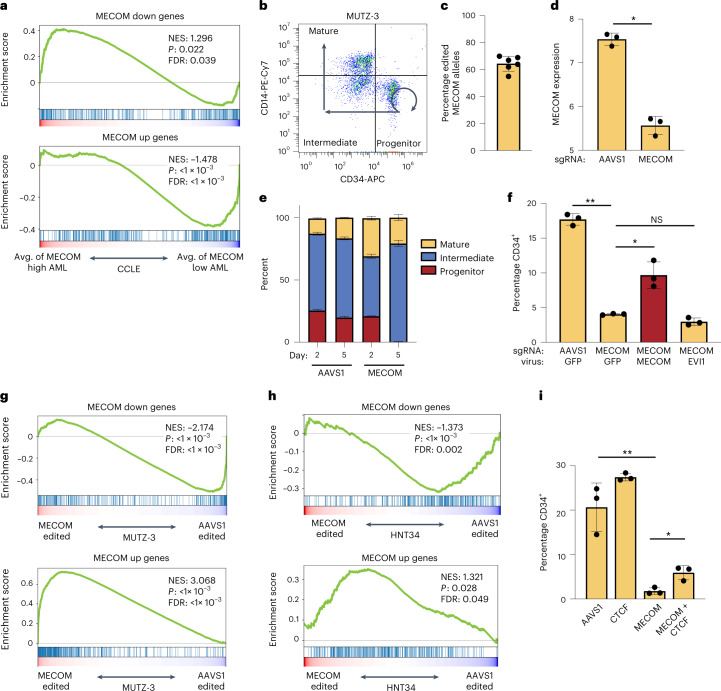
The MECOM gene regulatory network is indispensable in AML. **a**, GSEA of MECOM down genes and MECOM up genes in CCLE AML cell lines. AML cell lines were stratified by *MECOM* expression as in Extended Data Fig. 8a. *MECOM*-high AMLs show enrichment of MECOM down genes and depletion of MECOM up genes compared to *MECOM*-low AMLs. The K–S test was used to determine the significance of GSEA. **b**, FACS plot showing the immunophenotype of MUTZ-3 cells. CD34^+^CD14^−^ progenitors can self-renew (curved arrow) and undergo differentiation (straight arrows) into CD34^−^CD14^−^ intermediate promonocytes and ultimately CD34^−^CD14^+^ mature monocytes. **c**, *MECOM* editing in MUTZ-3 AML cells. Cells were collected on day 3 after nucleofection of CRISPR ribonucleoprotein (RNP) and the percent of modified alleles was determined by Sanger sequencing and ICE analysis; *n* = 6 biologically independent samples. Mean is plotted and error bar shows s.e.m. **d**, *MECOM* expression (log_2_ RPKM) in CD34^+^ MUTZ-3 cells. *MECOM* editing causes significant reduction in expression; *n* = 3 per group. Mean is plotted and error bars show s.e.m. Two-sided Student’s *t*-test was used. *P = 2 × 10^−4^. **e**, Myelomonocytic differentiation analysis of MUTZ-3 cells after CRISPR editing. Percent of cells within each subpopulation was measured by flow cytometry on days 2 and 5 after editing. *n* = 3 per group. Mean is plotted and error bars show s.e.m. **f**, Percentage of MUTZ-3 cells in CD34^+^CD14^−^ progenitor population after *MECOM* editing and viral rescue as determined by flow cytometry; *n* = 3 per group. Mean is plotted and error bars show s.e.m. Two-sided Student’s *t*-test was used. *P = 3.6 × 10^−2^, **P = 1.5 × 10^−3^. **g**,**h**, GSEA of MECOM network genes in MUTZ-3 cells (**g**) and HNT34 cells (**h**) after *MECOM* editing. *MECOM* perturbation in both AML cell lines results in enrichment of MECOM down genes and depletion of MECOM up genes. The K–S test was used to determine the significance of GSEA. **i**, Bar graphs of the rescue of CD34^+^ by dual *MECOM* and *CTCF* perturbation. MUTZ-3 AML cells underwent CRISPR editing with the sgRNA guides depicted on the *x* axis. Percent CD34^+^ cells were determined by FACS on day 4; *n* = 3 per group. Mean is plotted and error bars show s.e.m. Two-sided Student’s *t*-test was used. *P = 1.4 × 10^−2^, **P = 3.9 × 10^−3^. Source data

**Extended Data Fig. 8:**
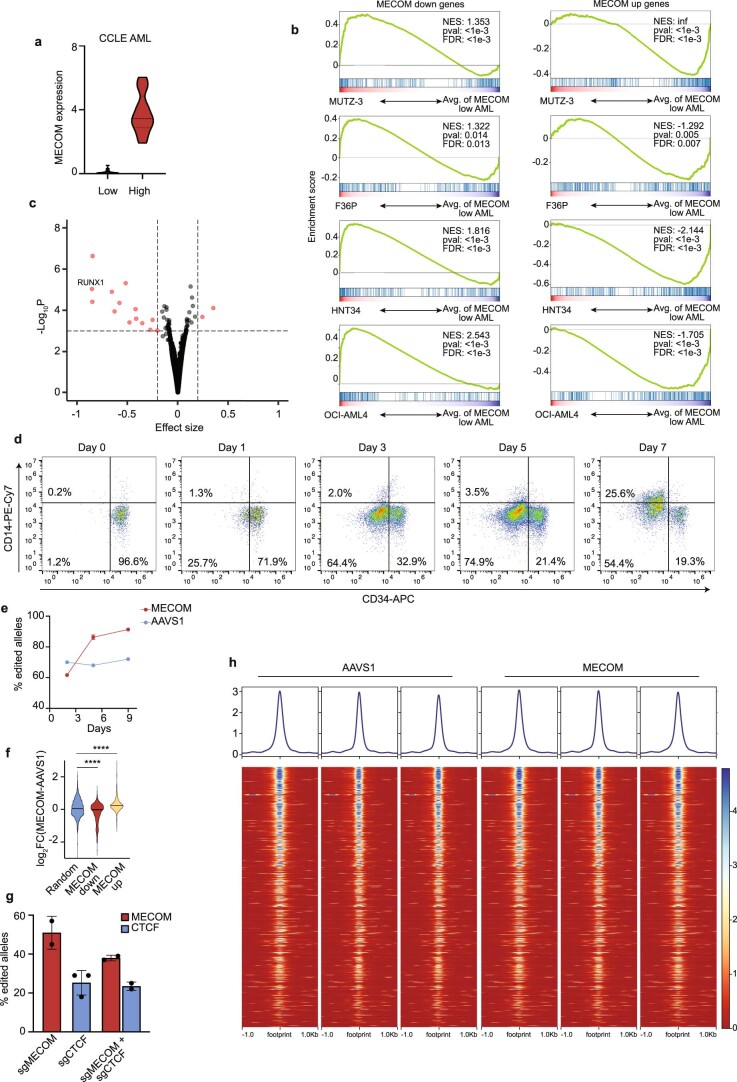
Evaluation of the MECOM gene network in high-risk AML. **(a)** Violin plots showing *MECOM* expression in AML samples from CCLE. AML samples were stratified by *MECOM* expression (log_2_ RPKM + 1). Low, <1 (*n* = 31); High≥1 (*n* = 13). Mean is plotted and dashed lines indicate quartiles. **(b)** GSEA of MECOM down genes and MECOM up genes in four AML cell lines with high MECOM expression compared to average expression in MECOM low AML cell lines. MUTZ-3, F36P, HNT34, and OCI-AML4 have enrichment of MECOM down genes and depletion of MECOM up genes. The Kolmogorov Smirnov (K-S) test was used to determine the significance of GSEA. **(c)** Volcano plot showing differential CRISPR dependencies of CCLE AMLs stratified by *MECOM* expression. Average CRISPR dependencies for the CCLE AML cohorts as defined in Extended Data Fig. 8a were determined using CERES and effect size was calculated by comparing dependency scores of *MECOM* high and *MECOM* low AMLs. Effect size of 0 indicates no difference in essentiality whereas negative effect size indicates higher essentially in *MECOM* high AML. Significance was calculated with Mann-Whitney U test. **(d)** FACS plots showing the differentiation of MUTZ-3 cells after CD34 selection. CD34^+^ MUTZ-3 cells were magnetically separated using the EasySep Human CD34 Positive Selection Kit II, cultured in MUTZ-3 media, and analyzed by flow cytometry at the indicated timepoints. **(e)** Time course of edited allele frequency in MUTZ-3 AML. Genotyping was performed in bulk MUTZ-3 cells following CRISPR editing at AAVS1 (blue) or MECOM (red). *n* = 3 biologically independent samples. Mean is plotted and error bars show s.e.m. Missing error bars are obscured by the icons. **(f)** Violin plot of differential gene expression in CD34^+^ MUTZ-3 cells following *MECOM* perturbation. MECOM down genes are significantly depleted and MECOM up genes are significantly enriched in *MECOM* edited samples compared to *AAVS1* edited samples, unlike a set of randomly selected genes. Two-sided Student *t*-test used. ****P = 1e-4. **(g)** Bar graphs of CRISPR editing frequencies in MUTZ-3 AML. Cells that underwent dual CRISPR perturbation of *MECOM* and *CTCF* had similar editing frequencies compared to single-edited cells. *n* = 2 biologically independent samples. Mean is plotted and error bars show s.e.m. **(h)** CTCF ChIP-seq in MUTZ3 cells after MECOM editing. MUTZ-3-Cas9 cells were transduced with sgMECOM lentivirus and cells were harvested on day 4 for ChIP-seq. There is significant CTCF binding to *cis*REs of MECOM down genes in MUTZ-3, but no differential binding after MECOM editing. Source data

Given the prognostic significance of MECOM network enrichment in AML, we sought to further study this network in AML cell lines. We examined 44 AML cell lines from the Cancer Cell Line Encyclopedia (CCLE) and stratified them based on *MECOM* expression (Extended Data Fig. 8a). We compared gene expression in *MECOM*-high compared to *MECOM*-low AML cell lines and found significant enrichment of MECOM down genes and depletion of MECOM up genes. (Fig. 8a). Comparison of gene expression in individual *MECOM*-high AML cell lines to the average expression in *MECOM*-low AML lines revealed highly significant MECOM network enrichment in MUTZ-3, F36P, HNT34 and OCI-AML4 cells (Extended Data Fig. 8b). We compared CRISPR dependencies of *MECOM*-high and *MECOM*-low AML cell lines and observed differential essentiality of RUNX1, consistent with our findings of potential cooperativity between RUNX1 and MECOM in regulating the HSC network genes (Extended Data Fig. 8c).

To validate the role of the MECOM network in an otherwise isogenic AML background, we performed CRISPR editing of *MECOM* in the MUTZ-3 AML cell line^45,46^. MUTZ-3 cells maintain a population of primitive CD34^+^ blasts in culture that can self-renew or differentiate into CD14^+^ monocytes (Fig. 8b and Extended Data Fig. 8d). *MECOM* editing in MUTZ-3 cells (Fig. 8c) resulted in significant reduction in *MECOM* expression level (Fig. 8d) and a loss of primitive CD34^+^ cells (Fig. 8e). Loss of progenitors after MECOM perturbation was accompanied by enrichment of edited *MECOM* alleles, as MECOM perturbed cells underwent greater expansion (Extended Data Fig. 8e). Maintenance of CD34^+^ cells was restored by lentiviral *MECOM* expression, but not lentiviral expression of the *EVI1* isoform (Fig. 8f), consistent with our rescue data from primary HSPCs (Extended Data Fig. 4e). RNA-seq of CD34^+^ progenitor MUTZ-3 cells after *MECOM* editing revealed significant depletion of MECOM down genes and significant enrichment of MECOM up genes (Fig. 8g, Extended Data Fig. 8f and Supplementary Table 7), Additionally, MECOM perturbation in HNT34 AML cells led to significant depletion of MECOM down genes and significant enrichment of MECOM up genes (Fig. 8h), revealing the conservation of this gene regulatory network in multiple AML contexts.

Because of the functional interaction between MECOM and CTCF in the transcriptional control of LT-HSC quiescence, we reasoned that the loss of MUTZ-3 progenitors following *MECOM* perturbation may also be dependent on CTCF. We performed dual CRISPR editing of *MECOM* and *CTCF* and observed partial rescue of the loss of CD34^+^ progenitors induced by *MECOM* perturbation alone (Fig. 8i). The more modest rescue of progenitors in the MUTZ-3 system compared to the LT-HSC model (Fig. 6i) may be a function of less efficient *CTCF* editing in MUTZ-3 cells (Extended Data Fig. 8g).

To evaluate binding of CTCF to the *cis*REs of MECOM network genes, we generated a Cas9 and GFP expressing MUTZ-3 cell line which, we infected with a lentivirus encoding an sgRNA targeting *AAVS1* or *MECOM* along with red fluorescent protein (RFP). We observed a gradual loss of CD34^+^ cells following MECOM sgRNA delivery and on day 4 after editing we examined CTCF binding in CD34^+^ MUTZ-3 progenitors by ChIP-seq before complete loss of CD34^+^ progenitors. In the AAVS1-treated samples, we observed strong CTCF binding in the *cis*REs of MECOM network genes that contain CTCF footprints (Extended Data Fig. 8h). There was no difference in CTCF binding after *MECOM* editing, suggesting that the co-regulation of MECOM network genes by CTCF is not due to differential CTCF chromatin occupancy in CD34^+^ MUTZ-3 cells, but may instead be due to differential cofactor interactions or chromatin looping. Collectively, these data reveal that the MECOM regulatory gene network co-regulated by CTCF is indispensable for AML progenitor maintenance.

## Discussion

A greater fundamental understanding of the transcriptional circuitry that enables human HSCs self-renewal holds considerable promise for future mechanistic studies of HSC function and therapeutic applications. For instance, with emerging advances in gene therapy and genome editing of HSCs, the ability to better maintain and manipulate these cells both ex and in vivo would be clinically beneficial^47^; however, the limitations in our molecular understanding of this regulatory process have hampered such efforts.

Here, we have taken advantage of a rare experiment of nature to illuminate fundamental transcriptional circuitry that is required for human HSC maintenance in vivo. We have followed up on the human genetic observation that *MECOM* haploinsufficiency results in early-onset bone marrow failure and by modeling this disorder in primary HSPCs, we show that the functional loss of HSCs is accompanied by alterations in a network of genes critical for HSC maintenance. The identification of this gene network highlights the need to couple rigorous functional assays that nominate cellular vulnerabilities with integrative genomic profiling and analyses. Our results demonstrate how subtle gene expression changes can translate into major defects in HSC maintenance and uncover additional regulators of HSCs that can be subject to systematic perturbation studies in the future.

Through integrative genomic analysis of this network, we have gained insights into critical gene targets and have elucidated cooperative interactions among hematopoietic TFs involved in HSC function. We identify an antagonistic role for CTCF in altering chromatin looping of MECOM network genes as the cells differentiate and validate this interaction by functional and molecular rescue, illuminating fundamental transcriptional circuitry required for human HSC maintenance. We also find that this very same network is co-opted in AMLs with poor prognosis. A notable finding is that the MECOM regulatory network serves as a better predictor of poor outcome than does *MECOM* expression itself, suggesting that some AMLs may augment MECOM function in a manner beyond expression changes. This will be an important area for future exploration. It is also notable that leukemias arising due to insertional mutagenesis following human gene therapy trials have resulted in activation of *MECOM*^48^. Clones with increased *MECOM* expression often have a long latency, but can result in a more aggressive disease course. Our finding that an HSC regulatory program is co-opted by increased *MECOM* expression may help explain these perplexing clinical observations. A deeper understanding of how such stem cell networks are utilized in malignant states may enable improved therapeutic approaches and provide opportunities to expand and manipulate non-malignant HSCs for therapeutic benefit.

## Methods

### Data reporting

No statistical methods were used to predetermine sample sizes but our sample sizes are similar to those reported in previous publications^16,23^,. Data distribution was assumed to be normal but this was not formally tested. Data collection and analysis were not performed blind to the conditions of the experiments. No animals or data points were excluded from analysis.

### Cell line and primary cell culture

HSPCs were purified from discarded UCB samples of healthy male or female newborns using the EasySep Human CD34 Positive Selection Kit II following pre-enrichment using the RosetteSep Pre-enrichment cocktail (Stem Cell Technologies) and mononuclear cell isolation on Ficoll-Paque (GE Healthcare) density gradient. Cells were cryopreserved for later use. Granulocyte colony-stimulating factor mobilized adult CD34^+^ HSPCs and were purchased (Fred Hutchinson Cancer Research Center). Thawed cells were cultured at 37 °C and 5% O_2_ in serum-free HSC medium consisting of StemSpan II medium (Stem Cell Technologies) supplemented with CC100 cytokine cocktail (Stem Cell Technologies), 100 ng ml^−1^ TPO (Peprotech) and 35 nM UM171 (Stem Cell Technologies). Confluency was maintained between 2 × 10^5^ and 1 × 10^6^ cells per ml.

MUTZ-3 cells (DSMZ) were cultured at 37 °C in α-MEM (Life Technologies) supplemented with 20% FBS, 20% conditioned medium from 5,637 cells (ATCC)^49^ and 1% penicillin/streptomycin. Confluency was maintained between 7 × 10^5^ and 1.5 × 10^6^ ml^−1^.

HNT34 cells (Creative Bioarrray) were cultured at 37 °C in α-MEM (Life Technologies) supplemented with 20% FBS, 20% conditioned medium from 5,637 cells (ATCC)^49^ and 1% penicillin/streptomycin. Confluency was maintained between 5 × 10^5^ and 1.5 × 10^6^ ml^−1^.

The 293T cells were cultured at 37 °C in DMEM (Life Technologies) supplemented with 10% FBS and 1% penicillin/streptomycin.

### Mouse model

NOD.Cg-*Kit*^W-41J^*Tyr*^+^*Prkdc*^scid^*Il2rg*^tm1Wjl^ (NBSGW) mice were obtained from the Jackson Laboratory (stock 026622)^21^. Littermates of the same sex were randomly assigned to experimental groups. NBSGW were interbred to maintain a colony of animals homozygous or hemizygous for all mutations of interest. The Institutional Animal Care and Use Committee at Boston Children’s Hospital approved the study protocol and provided guidance and ethical oversight

### CRISPR editing and analysis

Electroporation was performed on day 1 after thawing HSPCs using the Lonza 4D Nucleofector with 20 µl Nucleocuvette strips as described^23,50^. Briefly, the RNP complex was made by combining 100 pmol Cas9 (IDT) and 100 pmol modified sgRNA (Synthego) targeting MECOM (5′-CAAGGTCTGCAAACCTAACA-3′), AAVS1 (5′-GGGGCCACTAGGGACAGGAT-3′) or CTCF (5′-CAATTCTCCACTGGTCACAA-3′) and incubating at 21 °C for 15 min. Between 2 × 10^5^ and 4 × 10^5^ HSPCs resuspended in 20 µl P3 solution were mixed with RNP and underwent nucleofection with program DZ-100. For samples that underwent dual perturbation, total amounts of 100 pmol Cas9 and 100 mol sgRNA (50 pmol each guide) were used. Cells were returned to HSC medium and editing efficiency was measured by PCR at 48 h after electroporation, unless otherwise indicated. First, genomic DNA was extracted using the DNeasy kit (QIAGEN) or both DNA and RNA were extracted using the AllPrep DNA/RNA Mini kit (QIAGEN) according to the manufacturer’s instructions. Genomic PCR was performed using Platinum II Hotstart Mastermix (Thermo Fisher Scientific) and edited allele frequency was detected either by Sanger sequencing and analyzed by ICE (ice.syngthego.com) or NGS and analyzed with Crispresso2 (ref. ^51^). The following primer pairs were used: MECOM-ICE (forward: 5′-ACATCAACCCAGAATCAGAAAC-3′; reverse: 5′-GGAAAAGGAAGGCTGCAAAG-3′); MECOM-NGS (forward: 5′-AGAAATGTGAGTTCCATGCAAGA-3′; reverse: 5′-AGCAAATATCATTGTCAGACCTGT-3′); and CTCF (forward: 5′-CAGCGGATTCAGATGGGTAA-3′; reverse: 5′-TCACCGTTTTAGCCAGGATG-3′). The effect on MECOM mRNA after editing was detected by quantitative PCR with reverse transcription (qRT–PCR) using SYBR green (Bio-Rad) after cDNA synthesis with iScript (Bio-Rad).

MUTZ-3 cells were edited as above with the following modification: cells were resuspended in 20 µl SF solution and program EO-100 was used for electroporation.

### Viral constructs and transduction

*MDS* and *EVI1* cDNA were synthesized from mRNA of human HSPCs using the following primers: MDS (forward: 5′-CGTACTCGAGGCCGCCACCATGAGATCCAAAGGCAGGGCAA-3′; reverse: 5′-TACGGAATTCTCACTCCCATCCATAACTGGGGTCT-3′); and EVI1 (forward: 5′-CGTACTCG AGGCCGCCACCATGATCTTAGACGAATTTTACAATG-3′; reverse: 5′-TACGGAATTCTCATACGTGGCTTATGGACTGG-3′). *MECOM* cDNA was synthesized using MDS-F and EVI1-R primers. Wobble mutations were introduced to disrupt the sgRNA binding site using the following primers EVI1-F and wobble reverse (5′-GTGCCGAGTGAGATTCGCGGATCTAGGAAAAAT-3′) and wobble forward (5′-ATTTTTCCTAGATCCGCGAATCTCACTCGGCAC-3′) with EVI1-R, followed by overlap PCR of the two fragments. Primers included restriction enzyme sites to allow for cloning using EcoRI and XhoI into the HMD IRES–GFP backbone^52^.

The lentiviral pXPR_049 plasmid was obtained from the Genomics Perturbation Platform at the Broad Institute and RFP was cloned in place of the puromycin resistance gene. sgRNA sequences targeting *AAVS1* or *MECOM* as described above were cloned into pXPR_049-RFP using BsmBI. The lentiviral pXPR_104 plasmid encoding Cas9v3-2A-GFP was also obtained from the Broad Institute Genomics Perturbation Platform.

To produce lentivirus, approximately 24 h before transfection, 293T cells were seeded in 10-cm plates. Cells were co-transfected with 10 µg pΔ8.9, 1 µg VSVG and 10 µg HMD vector variant, Cas9–GFP or sgRNA–RFP using calcium phosphate. The medium was changed the following day and viral supernatant was collected 48 h after transfection, filtered with a 0.45-µm filter and concentrated by ultracentrifugation at 100,000*g* for 2 h at 4 °C.

For lentiviral rescue experiments, 24 h after CRISPR nucleofection, 1 × 10^5^ HSPCs were transduced at a multiplicity of infection (MOI) of 10, with HMD empty, MDS, EVI1 or MECOM virus in 12-well plates with 8 µg ml^−1^ of polybrene (Millipore), spun at 931*g* for 1.5 h at 21 °C and incubated in the viral supernatant overnight at 37 °C. Virus was washed off 16 h after infection.

MUTZ-3 cells were transduced at an MOI of 1 by spinfection at 1,455*g* for 1.5 h at 21 °C and were incubated in the viral supernatant overnight. Virus was washed off 16 h after infection. MUTZ-3 cells underwent viral transduction first, followed by CRISPR editing at 48 h after infection. MUTZ-3 or HNT34 cell lines expressing Cas9–GFP were generated by spinfection followed by GFP purification and subsequent spinfection with sgRNA–RFP virus and a second sorting for GFP^+^RFP^+^ cells.

### Transplantation assays

Non-irradiated NBSGW mice (between 4–8 weeks of age) were tail vein injected with UCB or adult CD34^+^ HSPCs (1–2 × 10^5^ cells) on day 3 after CRISPR editing. Peripheral blood was sampled monthly by retro-orbital sampling and animals were killed at 16 weeks for BM evaluation. Secondary transplantations were performed by directly transplanting 60% of total BM cells from primary recipients into secondary non-irradiated NBSGW recipients. Human chimerism was assessed by evaluation of the BMs of secondary recipients at 16 weeks by flow cytometry and *MECOM* sequencing.

### Flow cytometry and cell sorting

Cells were washed with PBS and stained with the following panel of antibodies to quantify and enrich for LT-HSCs: anti-CD34-PerCP-Cy5.5 (BioLegend, 343612), anti-CD45RA-APC-H7 (BD, 560674), anti-CD90-PECy7 (BD, 561558), anti-CD133-super bright 436 (eBioscience, 62-1338-42), anti-EPCR-PE (BioLegend, 351904) and anti-ITGA3-APC (BioLegend, 343808). LT-HSCs were defined by the following immunophenotype: CD34^+^CD45RA^−^CD90^+^CD133^+^ITGA3^+^EPCR^+^ (ref. ^16^). Three microliters of each antibody were used per 1 × 10^5^ cells in 100 µl. Total LT-HSC numbers were calculated as a product of the frequency of LT-HSCs by flow cytometry and total cell number in culture.

Human cell chimerism after xenotransplantation was determined by staining with anti-mouse CD45-FITC (BioLegend, 103108) and anti-human CD45-APC (BioLegend, 368512). Human cell subpopulations were detected in the BM of transplanted mice using the following antibodies: anti-human CD45-APC (BioLegend, 368512), anti-human CD3-Pacific Blue (BioLegend, 344823), anti-human CD19-PECy7 (BioLegend, 302215), anti-human CD11b-FITC (BioLegend, 301330), anti-human CD41a-FITC (eBioscience, 11-0419-42), anti-human CD34-Alexa 488 (BioLegend, 343518) and anti-human CD235a-APC (eBioscience, 17-9987-42). Aliquots were stained individually for CD34 and CD235 or with CD45 in conjunction with the other lineage-defining markers. Mice with human cell chimerism <2% in the BM were excluded from subpopulation analysis.

MUTZ-3 cells were stained with anti-CD34-APC (BioLegend, 343607) and anti-CD14-PECy7 (BioLegend, 367112).

Flow cytometric analyses were conducted on a BD LSRII, LSR Fortessa or Accuri C6 instruments and all data were analyzed using FlowJo software (v.10.8). FACS was performed on BD Aria and samples were collected in PBS containing 2% BSA and 0.01% Tween for immediate processing for sequencing on the 10x Genomics platform. Alternatively, single cells were sorted into PCR plates containing 5 µl Buffer RLT Plus (QIAGEN) with 1% BME and immediately frozen at −80 °C for G&T sequencing.

### Cell cycle analysis

For cell cycle analyses, on day 5 after CRISPR editing, cells were incubated with 5-ethynyl-2′-deoxyuridine (EdU) (Thermo Fisher Scientific, C10634) for 2 h, then fixed and permeabilized before cell surface staining as per the manufacturer’s recommendations. Multipotent progenitors were defined by the immunophenotype CD34^+^CD45RA^−^CD90^+^CD133^+^. Pegasus v.1.0 (https://github.com/klarman-cell-observatory/pegasus) in the Terra environment (https://app.terra.bio/#) was used to determine the expression of transcriptional signatures of cell cycle status of single LT-HSCs^53^.

Analysis of cell division was performed by carboxyfluorescein succinimidyl ester (CFSE) labeling (Thermo, Fisher Scientific C34554). At 24 h after CRISPR editing, cells were incubated with CFSE, washed and subjected to flow cytometric analysis to establish a baseline and again on day 5. Proliferation modeling was performed in FlowJo v.10.8.0. Replication index was calculated in FlowJo v.10.8.0 as the total number of divided cells / cells that underwent at least one division.

### Colony-forming unit cell assays

Three days after RNP electroporation, 500 CD34^+^ HSPCs were plated in 1 ml methylcellulose medium (H4034, Stem Cell Technologies) in triplicate unless otherwise noted. Primary colonies were counted after 14 d.

### 10x Genomics scRNA-seq

A suspension of 11,000 *AAVS1*-edited LT-HSCs and a suspension of 16,000 *MECOM*-edited LT-HSCs were loaded into two lanes of 10x RNA 3′ V3 kit (10x Genomics) according to the manufacturer’s guidelines. Libraries were constructed with distinct i7 barcodes, pooled in equal molecular concentrations and sequenced on one lane of Hiseq (Illumina) according to the manufacturer’s protocol. Briefly, 36 cycles were carried out for read1, 8 cycles for index1 and 90 cycles for read2, yielding ~15,000 reads per cell.

### Bulk RNA-seq

Total RNA was extracted using the RNeasy Micro kit (QIAGEN, 74004) or using the 2.2× RNAClean XP kit (Beckman, A63987) from ~1,000 cells sorted in 25 µl Buffer RLT Plus with 1% BME. Then we proceeded with the SmartSeq2 protocol from the reverse transcription step using 10 ng of RNA^54^. The whole transcriptome amplification step was set at ten cycles. The 15 bulk RNA libraries were pooled at equal molecular concentration and sequenced using the NextSeq550 High Output or Novaseq kit (Illumina) with 35 paired-end reads.

### Genome and transcriptome sequencing

Plates of sorted LT-HSCs were thawed from −80 °C on ice and an equal volume of prepared 2× Dynabeads was added. Samples were incubated at 72 °C for 1 min, then 56 °C for 2 min, followed by 10 min at 25 °C to allow for mRNA hybridization. Plates were placed on a magnet for 2 min and 8 µl of the supernatant containing genomic DNA (gDNA) was transferred into a new plate. Beads were washed twice in 10 µl of cold 1× Hybridization Buffer and once in PBS + RNase Inhibitor. All washes were transferred to the gDNA plate. Once PBS was removed, Dynabeads were immediately resuspended in 7.34 µl of SmartSeq2 Mix 1 and the plate was incubated at 80 °C for 3 min. The plate was immediately placed on the magnet and the supernatant containing mRNA was rapidly transferred into a new plate on ice. Then, 2.66 µl of SmartSeq2 Mix 2 was added. At this point, we proceeded with the SmartSeq2 protocol from the reverse transcription step^54^. The whole transcriptome amplification step was set at 23 cycles. gDNA which was present in the pooled supernatant/wash buffer was precipitated on DNA SPRI beads at a 0.6× ratio and eluted in 10 µl MDA Hyb buffer, denatured at 95 °C for 3 min and cooled on ice. Then 5 µl of Phi29 Mix was added and the mix was incubated at 45 °C for 8 h. The reaction was deactivated at 65 °C for 5 min. The MDA plate was stored at −20 °C. Eight plates of mRNA libraries were sequenced using the Nextseq550 high output kit (Illumina) with 35 paired-end reads according to the manufacturer’s recommendations. To genotype each cell based on *MECOM* editing status, *MECOM* from gDNA and whole transcriptome analysis was amplified by PCR and libraries were constructed, pooled and sequenced using the Miseq 300 cycle kit (Illumina) according to manufacturer’s protocol with 150 paired-end reads.

### ChIP-seq

Chromatin immunoprecipitation followed by sequencing (ChIP-seq) was performed on chromatin from 2×10^6^ CD34^+^MUTZ-3 after *MECOM* or *AAVS1* editing. Sorted cells were cross-linked with 1% methanol-free formaldehyde (Pierce Life Technologies, 28906), quenched with 0.125 M glycine and frozen at −80 °C and stored until further processing. ChIP reaction was performed with iDeal ChIP-seq kit for TFs (Diagenode, C01010055) with modifications of the manual detailed below. Lysed samples were sonicated using the E220 sonicator (Covaris, 500239) in microTUBE AFA Fiber Pre-Slit Snap-Cap tubes (Covaris, 520045) with settings for 200-bp DNA shearing. Sheared chromatin was immunoprecipitated with 2.5 μg CTCF antibody (Abcam, ab128873, RRID AB_11144295) or 2.5 μg IgG antibody (Diagenode, C15410206, RRID AB_2722554). Eluted and decross-linked DNA was purified with MicroChIP DiaPure columns (Diagenode, C03040001) and eluted in 30 μl of nuclease-free water. ChIP and input libraries for sequencing were prepared with ThruPLEX DNA-Seq kit (Takara, R400674) and DNA Single Index kit, 12S Set A (Takara, R400695). Size selection steps were performed with Magbio Genomics HighPrep PCR beads (Fisher Scientific, 50-165-6582). The libraries were sequenced at Broad Institute Genomic Services by using the Illumina NextSeq 500 platform and the 150-bp paired-end configuration to obtain at least 30 million reads per sample.

### Quantification and statistical analysis

#### Protein structure prediction

The MECOM sequence corresponding to amino acids 700–900 was submitted to the I-TASSER server for homology modeling^55^. The predicted structure of the zinc finger domain was rendered and visualized using PyMOL.

#### Bulk RNA data analysis

Fastq files demultiplexed by bcl2fastq from bulk RNA-seq run were uploaded to Terra and processed with the Cumulus pipeline for bulk RNA-seq^53^ to get gene counts and gene isoform matrices. Human reference genome GRCh38 and gene annotation reference Homo_sapiens.GRCh38.93.gtf were used in all the RNA analysis.

#### Single-cell RNA data analysis

BCL files generated by scRNA-seq were uploaded to Terra and processed with the Cumulus pipeline for 10x single-cell RNA data and SmartSeq2 (ref. ^53^) to get gene matrices. Human reference genome GRCh38 and gene annotation reference Homo_sapiens.GRCh38.93.gtf were used in all the RNA analyses. For 10x data, doublets were filtered out and cells that contained reads for 500 to 8,000 genes with the percent of mitochondrial genes <20% were included in the analysis; cells were not filtered based on unique molecular identifier counts. For SmartSeq2 data, Scanpy^56^ was used to integrate all plates and perform batch correction and normalization. Cells that contained reads for 2,000 to 20,000 genes with the percent of mitochondrial genes <20% were included. Genes expressed in at least 0.05% of cells were included. Scanorama^57^ was used for batch correction. SmartSeq2 and 10x data were integrated and batch correction was performed on donor, technology and process batch with a Python version of Harmony^58^. Celltypist^22^ was used to infer cell types with the Pan_Fetal_Human.pkl model.

#### *MECOM* genotyping in G&T data

*MECOM* editing was determined by CRISPResso2 (ref. ^51^). Genotyping from gDNA and from cDNA was combined for the same cell and cells that contained both an edited allele and a wild-type allele were defined as heterozygous. Genotyping annotation was integrated into gene matrix metadata.

#### Differential expression analysis

Differential expression analysis was performed by Seurat v.4.0 with the function FindMarkers pipeline in the 10x single-cell RNA data to compare *AAVS1*- and *MECOM*-edited LT-HSCs. The fold change threshold for significant gene expression was 0.05 on log_2_scale, ident.1 was *AAVS1*-edited cells, ident.2 was *MECOM*-edited cells and the test algorithm was MAST. Permutation analysis was performed by randomly assigning single cells to one of two groups irrespective of the initial experimental group and repeating differential expression analysis. One hundred independent permutations were performed.

#### Pseudobulk analysis

Raw counts from single LT-HSCs that passed the quality control from each experimental condition (*AAVS1* or *MECOM-*edited) were aggregated to generate pseudobulk data for each group. Genes that did not reach the detection ratio cutoff used in the single-cell differential gene expression discovery were removed from the pseudobulk analysis. Log_2_ fold change between groups was calculated and correlation with gene expression data from single cells was calculated by Spearman’s rank correlation.

#### HSC signatures in the Immune Cell Atlas

Pegasus was used to determine the expression of the HSC signature (CD34, HLF and CRHBP)^23^ in umbilical cord samples from the Immune Cell Atlas (https://data.humancellatlas.org/explore/projects/cc95ff89-2e68-4a08-a234-480eca21ce79).

#### Gene signature enrichment during hematopoiesis

We measured the enrichment of the MECOM down or MECOM up gene sets during hematopoiesis, using bulk RNA-seq datasets across 20 hematopoietic subpopulations^27^. The observed expression for the tested gene set in each cell type was calculated by taking the mean expression of genes in the list. We performed 1,000 permutations in which we sampled gene sets with the same number of genes as the tested gene set. The expected expression for permuted gene set in cell type was calculated by taking the mean expression of genes in the list. The enrichment for gene set in cell type was computed as follows:

$$z_{i,j} = \frac{{y_{i,\,j} - {\rm{mean}}(\,y_{i,\,j}^{(P)})}}{{{\rm{s.d.}}(\,y_{i,\,j}^{(P)})}}$$

where the mean and variance of $y_{i,j}^{(P)}$ are taken over all values of *P* $\left( {P \in (1,\,2,...,1,000)} \right.$.

#### Gene set enrichment analysis

We used GSEApy (https://github.com/zqfang/GSEApy) for all GSEA analyses to determine the enrichment of MECOM network genes following *MECOM* editing and rescue and in the TCGA and CCLE datasets that were stratified based on *MECOM* expression or overall survival. Significant enrichment of the gene set was determined using a *t*-test for MECOM rescue in LT-HSCs and MUTZ-3 cells and diff_of_classes for TCGA analyses. Genes from CCLE data were preranked by determining mean expression for each gene in AML-high and AML-low cohorts and calculating log_2_ fold change. GSEA was performed using 1,000 permutations to determine significance.

#### Development of HemeMap

A detailed description is provided in the Supplementary Note^59–65^.

#### ChIP-seq data analysis

The raw ChIP-seq data^35^ for the binding sites of hematopoietic TFs FLI1, GATA2 and RUNX1 in human CD34^+^ HSPCs, were downloaded and processed. The paired-end reads were trimmed and aligned to hg19 reference genome using Trimmomatic and Bowtie2, respectively. MACS2 (ref. ^66^) was used for peak calling with the default narrow peak setting. Genomic tracks were generated from BAM files using counts per million mapped reads normalization to facilitate comparison between tracks. The processed CTCF ChIP-seq data from HSPCs and differentiated hematopoietic lineages were obtained from a previous study^38^. To determine the significance of the enrichment of TF occupancy within *cis*REs of MECOM network genes, a permutation test was performed. For each TF, we calculated the number of *cis*REs overlapping with ChIP-seq peaks. The expected distribution of overlapping *cis*REs was generated by 1,000 permutations of an equal number of TF peaks across the genome. The presence of TF peaks in *cis*REs were counted and the Venn plot was generated by the web app BioVenn (https://www.biovenn.nl). The enrichment of CTCF signal on the footprints was performed using deepTools software^67^. We used a Wilcoxon signed-rank test to evaluate the differences of normalized CTCF signals on footprints between HSPCs and other terminal blood cells, namely erythroid cells, T cells, B cells and monocytes.

#### CTCF-mediated loop enrichment analysis

A set of 7,358 representative chromatin interactions in hematopoietic cells was identified from a high-resolution Hi-C map of OCI-AML2 cells as previously described^37^. The loops whose anchors overlap with *cis*REs of MECOM down genes were extracted for further analysis. The CTCF-mediated loops (at least one of the anchors containing a CTCF footprint) and non-CTCF-mediated loops (anchors without CTCF footprint) were identified separately. The Low-C data of chromatin looping in LT- and ST-HSC were normalized by Knight–Ruiz balanced interaction frequencies at a resolution of 25 Kb. We used Juicer to perform aggregate peak analysis^36^ to test for enrichment of loops within the Low-C data from LT-HSCs and ST-HSCs. Loops containing genes were identified by the genes within the genomic domains between loop anchors.

### Analysis of primary AML patient data

#### Included studies

Three study cohorts were included in the survival analyses. We downloaded RNA-seq V2 expression data and corresponding clinical outcomes from the TCGA LAML^39^ cohort from cBioPortal (https://www.cbioportal.org/study/summary?id=laml_tcga_pub) for 173 patients with AML. The same was conducted for the BEAT AML cohort for 430 patients (https://www.cbioportal.org/study/summary?id=aml_ohsu_2018)^40^. In addition, the TARGET dataset was downloaded for 440 pediatric patients with AML (https://www.cbioportal.org/study/summary?id=aml_target_2018_pub)^41^. To gain maximal insight, adult datasets (TCGA and BEAT) were combined, with subsequent adjustments in analyses to account for study specific features. The only pediatric data used were from the TARGET dataset. The results published here are in part based upon data generated by the Therapeutically Applicable Research to Generate Effective Treatments (https://ocg.cancer.gov/programs/target) initiative, phs000218. The data used for this analysis are available at https://portal.gdc.cancer.gov/projects.

#### Derivation of variables of interest

A detailed description is provided in the Supplementary Note.

#### Survival analyses

KM curves were constructed demonstrating survival for each cohort (adult and pediatric) and variables (*MECOM* expression, MECOM network enrichment score, MECOM network enrichment (categorical), LSC17 and clinical risk score). For continuous variables, to appreciate survival differences in the variable in this way, KM curves were stratified by thresholding on the optimum threshold determined by Youden’s *J* statistic, maximizing both sensitivity and specificity of the metric. Follow-up time was truncated at 2,500 d for the pediatric cohort (thereby including *n* = 350, 79.5% of all complete cases) and at 1,500 d for the adult cohort (thereby including *n* = 513, 83.8% of all complete cases) for this and subsequent analyses to limit the issue of data sparsity at very late event time points. KM curves were constructed in R using survival and ggsurvplot packages.

HRs and 95% CI of death were determined from Cox proportional hazards models. These were created for each variable, correcting for contributing study in the adult group. This allowed assessment of continuous variables at their full spectrum. This also allowed for assessment of association of MECOM down network enrichment with mortality, independent of existing clinical approaches such as the clinical risk score and LSC17. Corrected models for age and sex were created and marginal hazard of mortality was derived and displayed graphically by different ages. The R packages’ coxph, survival, rms and ggeffects were used.

For analysis of AML cells from the CCLE database, we downloaded RNA-seq and CRISPR dependency data from the Cancer Dependency Map (https://depmap.org)^68^. We stratified the cohort based on *MECOM* expression (*MECOM*-low, log_2_(RPKM + 1) < 1; *MECOM*-high, log_2_(RPKM + 1) ≥ 1). Differential essentiality was determined by subtracting the CERES gene effect score of *MECOM*-high and *MECOM*-low AML samples. A negative value indicates stronger essentiality in *MECOM*-high AML.

### Reporting summary

Further information on research design is available in the Nature Portfolio Reporting Summary linked to this article.

## Online content

Any methods, additional references, Nature Portfolio reporting summaries, source data, extended data, supplementary information, acknowledgements, peer review information; details of author contributions and competing interests; and statements of data and code availability are available at 10.1038/s41590-022-01370-4.

## Supplementary information

Supplementary Information Supplementary Methods.

Reporting Summary

Supplementary Table 1 Supplementary Tables 1–7.

## Data Availability

Summary statistics from RNA-seq studies are available in Supplementary Tables 2,3 and 7. HemeMap correlation data are available in Supplementary Tables 4 and 5. All sequencing data are deposited in National Center for Biotechnology Information Gene Expression Omnibus under Super Series GSE175521, including GSE175515 for MUTZ-3 and primary human CD34^+^ LT-HSPC bulk RNA-seq; GSE175516 for LT-HSPC 10x Genomics single-cell RNA-seq data; GSE175518 for primary human CD34^+^ LT-HSPC Amplicon-seq data; GSE175520 for primary human CD34^+^ LT-HSPC SmartSeq2 data; GSE214399 for CTCF in MUTZ-3 ChIP-seq data; and GSE216225 for F36P, HNT34 and primary human CD34^+^ HSPC bulk RNA-seq data and HSPC 10x Genomics scRNA-seq data. Publicly available AML gene expression data were downloaded from the following links and analyzed as described in the Methods: TCGA LAML (https://www.cbioportal.org/study/summary?id=laml_tcga_pub), TARGET AML (https://www.cbioportal.org/study/summary?id=aml_target_2018_pub) and BEAT AML (https://www.cbioportal.org/study/summary?id=aml_ohsu_2018). Source data are provided with this paper.

## Source data

Source Data Fig. 1 Statistical source data.
Source Data Fig. 2 Statistical source data.
Source Data Fig. 4 Statistical source data.
Source Data Fig. 6 Statistical source data.
Source Data Fig. 8 Statistical source data.
Source Data Extended Data Fig. 1 Statistical source data.
Source Data Extended Data Fig. 2 Statistical source data.
Source Data Extended Data Fig. 3 Statistical source data.
Source Data Extended Data Fig. 4 Statistical source data.
Source Data Extended Data Fig. 6 Statistical source data.
Source Data Extended Data Fig. 7 Statistical source data.
Source Data Extended Data Fig. 8 Statistical source data.

## Code availability

Source data for reproducing results of this study are available on GitHub (https://github.com/sankaranlab/mecom_var).
