# Supplementary material for: A genetic disorder reveals a hematopoietic stem cell regulatory network co-opted in leukemia
Source: Nat Immunol. 2022 Dec 15;24(1):69–83. doi: 10.1038/s41590-022-01370-4 (PMC9810535; doi:10.1038/s41590-022-01370-4)
Supplement: Supplementary file 1 — Supplementary Methods. [file 41590_2022_1370_MOESM1_ESM.pdf]

nature immunology

Article

https://doi.org/10.1038/s41590-022-01370-4

A genetic disorder reveals a hematopoietic stem cell regulatory network co-opted in leukemia

In the format provided by the authors and unedited

Supplementary methods

Development of HemeMap

***1.* Construction of HSC specific regulatory network.** *Cis*-regulatory elements (*cis*REs) govern gene expression via functional interaction with gene promoter directly or indirectly mediated by other *cis*REs[59]. To decipher the transcriptional regulation underlying human hematopoiesis, we developed a computational approach called HemeMap, by leveraging a set of multi-omic data in different hematopoietic populations to define *cis*REs, their target genes and their putative regulatory activity throughout hematopoiesis.

2. Identification of *cisREs*. To identify the putative *cis*REs in the human hematopoiesis, we used a consensus peak set of ATAC-seq data across 18 cell types across the hematopoiesis, similar to that which we employed in our previous studies[21,29]. The peaks were called using MACS2[60] for each cell type and uniformly resized to a width of 500 bp centered on the peak summits, then filtered by the ENCODE hg19 blacklist (https://www.encodeproject.org/annotations/ENCSR636HFF/). Peaks uniquely occurring in a particular cell type, i.e. non-overlapping with peaks from other cell types, were retained. For the peaks overlapping in two or more cell types, we compared them iteratively and kept the most significant peak. The remaining peaks were further filtered if they overlapped with gene promoters, which were defined as 500 bp regions around transcription start sites (TSS) of protein coding genes. The *cis*REs from the entire hematopoietic catalog consisted of 432,428 consensus accessible peaks and 18,492 gene promoters.

3. Identification of direct interactions. To find the interactions between genes and *cis*REs, we searched for all possible connections between gene and *cis*REs within 500 kb of gene TSS. We used two criteria to define the interactions which the *cis*RE could exert a direct effect on gene regulation: (1) experimental evidence of physical interaction in three-dimensional space or (2) a strong correlation between chromatin accessibility of *cis*RE and target gene expression. To this end, we annotated the nominated links to assess whether *cis*REs and target genes are spatially colocalized (i.e. in a chromatin loop). A published dataset spanning 17 hematopoietic cell types of promoter capture Hi-C (PCHi-C) data was used[28] and only loops with CHiCAGO score > 5 were considered. Next, we computed ATAC-seq reads falling within *cis*REs across the hematopoietic cell populations and performed normalization using the count per million (CPM) method. We calculated the Pearson correlation coefficient between chromatin accessibility of *cis*REs and gene expression across 16 hematopoietic cell types for each possible interaction pair. To determine the significance, we applied Fisher's z transformation to correlation coefficients. All the interactions with > 0.345 (equivalent to P value < 0.05) were kept. Finally, the nominated links that passed either of these two analyses were retained and a total of 1,218,933 direct interactions were identified.

4. Identification of indirect interactions. A gene regulatory network is established by a chain of *cis*REs which connect to the target though direct or indirect manners. Previous studies[35] reported that a number of cooperative *cis*REs could associate with the promoter and other *cis*REs related in multi-way contacts in chromatin loops. Co-accessible chromatin has been reported to be highly connected and functionally related, which is useful to evaluate the connectivity between *cis*REs. To identify the indirect interactions, we first computed the co-accessibility across 18 cell types between *cis*REs (not including gene promoters) whose genomic distance less than 500 kb. By using the Pearson correlation measurement and Fisher's z transformation as described above, the co-accessible *cis*RE-*cis*RE links with a correlation coefficient > 0.362 (equivalent to P value < 0.05) were selected. Next, to find the shortest path between a *cis*RE and its target promoter, we constructed a regulatory network using the direct gene-*cis*RE interactions and co-accessible

*cis*RE-*cis*RE links, and found the shortest paths between *cis*REs and genes in this network. Specifically, the network was built using the igraph R package with gene-*cis*RE interactions and *cis*RE-*cis*RE links. Dijkstra's algorithm is designed for searching for the shortest paths between nodes in a graph. In our network, we used this method to find all the potential indirect interactions mediated by the *cis*REs that have direct gene interactions identified in the first step of our analysis. Given that a smaller weight indicates a greater chance in participating in the shortest path found by the Dijkstra's method, we added the weight to each edge in the network: weight of a pseudo number of 1e-5 for direct gene-*cis*RE interactions and for *cis*RE-*cis*RE links, respectively. All of the gene-*cis*RE pairs that did not pass the direct interaction identification were analyzed by Dijkstra's method. The *cis*REs were filtered out if they were not linked to any gene. In total, 4,315,536 interaction pairs are included in HemeMap.

5. HSC specific regulatory network. To define the strengths of *cis*-regulatory interactions in each cell type, we calculated the HemeMap score by using the geometric mean of ATAC-seq signal over all the *cis*REs involved in each interaction to avoid potential bias introduced by the outliers. To get the HSC-specific regulatory network, we used the cumulative Chi-Square distribution to determine an interaction strength threshold of greater than 8.91 which filtered out 95% of the interactions. The remaining interactions were used to build an HSC-specific regulation network containing 12,808 genes and 372,491 *cis*REs.

6. Benchmarking of HemeMap. To validate the activity of *cis*REs, we used different epigenomic marks including those present active enhancers (H3K4me1 and DNase I) and those present repressive domains (H3K27me3) of HSPCs from the Roadmap Consortium[31]. We also employed genomic interaction data of HiC[30] and predicted interactions in HSPCs from the Roadmap Consortium[31] to validate that the nominated interactions are active in the HSPC compartment.

De novo motif discovery

To explore the MECOM mediated regulatory network, we retrieved all of the *cis*REs associated with MECOM network genes identified as differentially expressed after *MECOM* editing. We used the 200 bp sequences centered on *cis*REs, i.e. the genomic regions around summits of peaks or TSS, as input for the *de novo* motif discovery analysis. The MEME suite[61] was used and all the motifs with reported *E* value < 1e-20 were collected from results of DREME[62] and MEME. Similarity of *de novo* motifs and the putative TF motifs from a comprehensive collection of 401 human TFBS models (HOCOMOCO V11)[63] was performed using Tomtom[64]. We also correlated the similarity of the ETS family motif identified via *de novo* motif discovery with the EVI1 binding motif from a published dataset[11] by calculating the Pearson correlation coefficient of the Position Frequency Matrix (PFM) of the two motifs using universal motif R package.

TF Footprinting analysis

A TF footprint is a particular pattern of Tn5 enzyme cleavage sites generated by ATAC-seq data that enables analysis of chromatin occupancy at the base-pair resolution. There is a depletion of cleavage events at the specific site of TF binding on open chromatin, which allowed for the identification of TF binding events with the consensus motifs of interest from the *de novo* motif discovery analysis[65]. For each *de novo* motif, including ETS, RUNX, JUN, KLF, CTCF and GATA, we scanned all of the consensus motif sequences that occur within the *cis*REs in MECOM-mediated regulatory network using the software FIMO[66] with default parameters, except for a significance threshold of 5e-4. To create a nucleotide resolution cleavage frequency profile for each TF, we used *make_cut_matrix* function (https://github.com/Parkerlab/atactk) to count the Tn5 enzyme cleavage frequency at the recognized motif sites and their flanking +/- 250 bp sequences, using ATAC-seq data from HSCs. Then, we used CENTIPEDE[67] to build an

unsupervised Bayesian mixture model with the cleavage frequency profile to generate a posterior probability value for each motif instance. A motif instance was considered a footprint that is bound by a particular TF when the posterior probability score was greater than 0.95. The plot of cleavage frequency around the footprints was created by aggregating both strands using a custom R script.

Footprint co-occurrence analysis

To explore how these TFs cooperate with each other via combinatorial binding on the *cis*REs of MECOM network genes, we evaluated the co-occurrence of the TF footprints. Specifically, a hypergeometric test was employed to determine the statistical significance of co-occurrence of two different footprints, as depicted by the following equation:

$$\mathrm{Prob}(\mathrm{C} \geq c') = \sum_{i=c'}^{\min(f1,f2)} \frac{\binom{f1}{i}\binom{N-f1}{f2-i}}{\binom{N}{f2}}$$

where N is the total number of *cis*REs, $f1$ and $f2$ are the number of *cis*REs containing footprints of each of the two tested TFs, respectively. *P* value measuring the significance of enrichment is the tail probability of observing c' or more *cis*REs containing both TF footprints.

Derivation of variables of interest

We log2-transformed the TCGA normalized read counts and stratified the cohort based on MECOM expression (MECOM low, log2(RPKM+1)<4; MECOM high, log2(RPKM+1)≥4). LSC17 score was calculated as follows: (*DNMT3B* × 0.0874) + (*ZBTB46* × −0.0347) + (*NYNRIN* × 0.00865) + (*ARHGAP22* × −0.0138) + (*LAPTM4B* × 0.00582) + (*MMRN1* × 0.0258) + (*DPYSL3* × 0.0284) + (*KIAA0125* × 0.0196) + (*CDK6* × −0.0704) + (*CPXM1* × −0.0258) + (*SOCS2* × 0.0271) + (*SMIM24* × −0.0226) + (*EMP1* × 0.0146) + (*NGFRAP1* × 0.0465) + (*CD34* × 0.0338) + (*AKR1C3* × −0.0402) + (*GPR56* × 0.0501)[43]. For each of the three included studies, the expression of each gene in each individual sample was compared to the mean expression in the pertaining study cohort. GSEA (as described previously) was performed to determine the enrichment or depletion of MECOM down genes in each sample compared to the mean. A sample was determined to have enrichment of MECOM down genes if the Normalized Enrichment Score >0 and p-value <0.05, depletion of MECOM down genes if NES <0 and p-value <0.05, or unchanged MECOM down genes if p-value >0.05. In addition, the normalized enrichment score was studied as a continuous measure of MECOM network status. Clinical risk scoring was provided in tables by each of the studies based on the National Comprehensive Cancer Network criteria, and in this analysis are labelled as Adverse, Intermediate and Favorable for consistency.
