# Supplementary material for: A genetic disorder reveals a hematopoietic stem cell regulatory network co-opted in leukemia
Source: Nat Immunol. 2022 Dec 15;24(1):69–83. doi: 10.1038/s41590-022-01370-4 (PMC9810535; doi:10.1038/s41590-022-01370-4)
Supplement: Supplementary file 2 — Reporting Summary [file 41590_2022_1370_MOESM2_ESM.pdf]

nature portfolio

Corresponding author(s): Vijay G. Sankaran

Last updated by author(s): October 21, 2022

Reporting Summary

Nature Portfolio wishes to improve the reproducibility of the work that we publish. This form provides structure for consistency and transparency in reporting. For further information on Nature Portfolio policies, see our Editorial Policies and the Editorial Policy Checklist.

Statistics

For all statistical analyses, confirm that the following items are present in the figure legend, table legend, main text, or Methods section.

n/a	Confirmed	
☐	☒	The exact sample size (*n*) for each experimental group/condition, given as a discrete number and unit of measurement
☐	☒	A statement on whether measurements were taken from distinct samples or whether the same sample was measured repeatedly
☐	☒	The statistical test(s) used AND whether they are one- or two-sided<br>*Only common tests should be described solely by name; describe more complex techniques in the Methods section.*
☐	☒	A description of all covariates tested
☐	☒	A description of any assumptions or corrections, such as tests of normality and adjustment for multiple comparisons
☐	☒	A full description of the statistical parameters including central tendency (e.g. means) or other basic estimates (e.g. regression coefficient) AND variation (e.g. standard deviation) or associated estimates of uncertainty (e.g. confidence intervals)
☐	☒	For null hypothesis testing, the test statistic (e.g. F, t, r) with confidence intervals, effect sizes, degrees of freedom and P value noted<br>*Give P values as exact values whenever suitable.*
☐	☒	For Bayesian analysis, information on the choice of priors and Markov chain Monte Carlo settings
☐	☒	For hierarchical and complex designs, identification of the appropriate level for tests and full reporting of outcomes
☐	☒	Estimates of effect sizes (e.g. Cohen's *d*, Pearson's *r*), indicating how they were calculated

Our web collection on statistics for biologists contains articles on many of the points above.

Software and code

Policy information about availability of computer code

Data collection	No software was used for data collection; data were already collected, de-identified and provided to researchers for use.
Data analysis	The majority of the analyses were performed in R (version 3.6.3) and the specific packages and reproducible code are available on GitHub (https://github.com/sankaranlab/mecom_var). We also used Prism (v8.4), Pegasus (1.0), Python (3.7.7), PyMOL, FIMO, MACS2 and FlowJo (10.8).

For manuscripts utilizing custom algorithms or software that are central to the research but not yet described in published literature, software must be made available to editors and reviewers. We strongly encourage code deposition in a community repository (e.g. GitHub). See the Nature Portfolio guidelines for submitting code & software for further information.

Data

Policy information about availability of data

All manuscripts must include a data availability statement. This statement should provide the following information, where applicable:

- Accession codes, unique identifiers, or web links for publicly available datasets
- A description of any restrictions on data availability
- For clinical datasets or third party data, please ensure that the statement adheres to our policy

Summary statistics from RNA sequencing studies are available in Supplementary tables 2, 3, and 7. HemeMap correlation data are available in Supplementary tables 4, and 5. All sequencing data are deposited in National Center for Biotechnology Information Gene Expression Omnibus under Super Series GSE175521, including GSE175515 for MUTZ-3 and primary human CD34+ LT-HSPC bulk RNA-Seq; GSE175516 for LT-HSPC 10X Genomics single cell RNA-Seq data; GSE175518 for primary human CD34+ LT-HSPC Amplicon-Seq data; GSE175520 for primary human CD34+ LT-HSPC Smart-Seq2 data; GSE214399 for CTCF in MUTZ-3 ChIP-Seq data; and GSE216225 for F36P, HNT34 and primary human CD34+ HSPC bulk RNA-Seq data and HSPC 10x Genomics single cell RNA-seq data. Publicly available AML gene

expression data were downloaded from the following links and analyzed as described in the Methods section: TCGA LAML (https://www.cbioportal.org/study/summary?id=laml_tcga_pub), TARGET AML (https://www.cbioportal.org/study/summary?id=aml_target_2018_pub), and BEAT AML (https://www.cbioportal.org/study/summary?id=aml_ohsu_2018).

Field-specific reporting

Please select the one below that is the best fit for your research. If you are not sure, read the appropriate sections before making your selection.

☒ Life sciences ☐ Behavioural & social sciences ☐ Ecological, evolutionary & environmental sciences

For a reference copy of the document with all sections, see nature.com/documents/nr-reporting-summary-flat.pdf

Life sciences study design

All studies must disclose on these points even when the disclosure is negative.

Sample size	For all experiments, at least n=3 biological replicates were utilized for each condition and are clearly labeled in the figure legends and methods sections. No statistical methods were used to predetermine sample sizes but our sample sizes are similar to those reported in previous publications.
Data exclusions	No data were excluded.
Replication	All attempts at replication of these experimental data, including primary cell data from samples collected from different healthy donors, were successful. All in vitro functional assays were performed at least twice with cells derived from different donors and were successful.
Randomization	Inherent donor variability in experiments using primary human samples was controlled by ensuring that the control and experimental groups for any individual experiment were generated using cells from the same donor which were pooled and then aliquoted into the control and experimental groups. For in vivo studies, equal numbers of male and female mice were used in control and experimental groups. When multiple litters of mice were used, the litters were evenly split and distributed between control and experimental groups.
Blinding	Data collection and analysis were not performed blind to the conditions of these in vitro and mouse in vivo biological studies, similar to what has been done in previous similar studies as knowledge of this information was essential to conduct these studies.

Reporting for specific materials, systems and methods

We require information from authors about some types of materials, experimental systems and methods used in many studies. Here, indicate whether each material, system or method listed is relevant to your study. If you are not sure if a list item applies to your research, read the appropriate section before selecting a response.

Materials & experimental systems

n/a	Involved in the study	
☐	☒	Antibodies
☐	☒	Eukaryotic cell lines
☒	☐	Palaeontology and archaeology
☐	☒	Animals and other organisms
☒	☐	Human research participants
☒	☐	Clinical data
☒	☐	Dual use research of concern

Methods

n/a	Involved in the study	
☐	☒	ChIP-seq
☐	☒	Flow cytometry
☒	☐	MRI-based neuroimaging

Antibodies

Antibodies used

anti-CD34-PerCP-Cy5.5 (Biolegend, 343612, clone 561)
anti-CD45RA-APC-H7 (BD, 560674, clone HI100)
anti-CD90-PECy7 (BD, 561558, clone 5E10)
anti-CD133-super bright 436 (Ebioscience, 62-1338-42, clone TMP4)
anti-EPCR-PE (Biolegend, 351904, clone RCR-401)
anti-ITGA3-APC (Biolegend, 343808, clone ASC-1)
anti-mouse CD45-FITC (Biolegend, 103108, clone 30-F11)
anti-human CD45-APC (Biolegend, 368512, clone 2D1)
anti-human CD3-Pacific Blue (Biolegend, 344823, clone SK7)
anti-human CD19-PECy7 (Biolegend, 302215, clone HIB19)
anti-human CD11b-FITC (Biolegend, 301330, clone ICRF44)
anti-human CD41a-FITC (Ebioscience, 11-0419-42, clone HIP8)
anti-human CD34-Alexa 488 (Biolegend, 343518, clone 581)
anti-human CD235a-APC (Ebioscience, 17-9987-42, clone HIR2)
anti-CD34-APC (Biolegend, 343607, clone 561)

	anti-CD14-PECy7 (Biolegend, 367112, clone 63D3)<br>anti-CTCF (abcam, ab128873, RRID:AB_11144295)<br>anti-IgG (Diagenode, C15410206, RRID: AB_2722554)<br>Three microliters of each antibody were used per 1e5 cells in 100µl unless otherwise specified.
Validation	anti-CD34-PerCP-Cy5.5 (Biolegend, 343612, clone 561): antibody validated by FACS analysis of stained primary human hematopoietic stem and progenitor cells<br>anti-CD45RA-APC-H7 (BD, 560674, clone HI100): antibody validated by FACS analysis of stained primary human hematopoietic stem and progenitor cells<br>anti-CD90-PECy7 (BD, 561558, clone 5E10): antibody validated by FACS analysis of stained primary human hematopoietic stem and progenitor cells<br>anti-CD133-super bright 436 (Ebioscience, 62-1338-42, clone TMP4):antibody validated by FACS analysis of stained primary human hematopoietic stem and progenitor cells<br>anti-EPCR-PE (Biolegend, 351904, clone RCR-401): antibody validated by FACS analysis of stained primary human hematopoietic stem and progenitor cells<br>anti-ITGA3-APC (Biolegend, 343808, clone ASC-1): antibody validated by FACS analysis of stained primary human hematopoietic stem and progenitor cells<br>anti-mouse CD45-FITC (Biolegend, 103108, clone 30-F11): antibody validated by FACS analysis of stained primary mouse peripheral blood mononuclear cells<br>anti-human CD45-APC (Biolegend, 368512, clone 2D1): antibody validated by FACS analysis of stained primary human peripheral blood mononuclear cells<br>anti-human CD3-Pacific Blue (Biolegend, 344823, clone SK7): antibody validated by FACS analysis of stained primary human peripheral blood mononuclear cells<br>anti-human CD19-PECy7 (Biolegend, 302215, clone HIB19): antibody validated by FACS analysis of stained primary human peripheral blood mononuclear cells<br>anti-human CD11b-FITC (Biolegend, 301330, clone ICRF44): antibody validated by FACS analysis of stained primary human peripheral blood mononuclear cells<br>anti-human CD41a-FITC (Ebioscience, 11-0419-42, clone HIP8): antibody validated by FACS analysis of stained primary human hematopoietic stem and progenitor cells<br>anti-human CD34-Alexa 488 (Biolegend, 343518, clone 581): antibody validated by FACS analysis of stained primary human hematopoietic stem and progenitor cells<br>anti-human CD235a-APC (Ebioscience, 17-9987-42, clone HIR2): antibody validated by FACS analysis of stained primary human peripheral blood cells<br>anti-CD34-APC (Biolegend, 343607, clone 561): antibody validated by FACS analysis of stained MUTZ-3 cells<br>anti-CD14-PECy7 (Biolegend, 367112, clone 63D3): antibody validated by FACS analysis of stained MUTZ-3 cells<br>anti-CTCF (abcam, ab128873, RRID:AB_11144295): validated by chromatin immunoprecipitation followed by targeted qPCR for known binding loci.<br>anti-IgG (Diagenode, C15410206, RRID: AB_2722554): validated no chromatin immunoprecipitation by DNA quantification and targeted qPCR analyese

Eukaryotic cell lines

Policy information about cell lines

Cell line source(s)	MUTZ-3 cells (DSMZ), 5637 cells (ATCC), 293T (ATCC), HNT34 (Creative Bioarray)
Authentication	Cell lines were purchased directly from the suppliers as listed and validated by STR analysis as appropriate.
Mycoplasma contamination	All cell lines were routinely tested for mycoplasma contamination and were negative.
Commonly misidentified lines<br>(See ICLAC register)	None of the cell lines are listed in the ICLAC database

Animals and other organisms

Policy information about studies involving animals; ARRIVE guidelines recommended for reporting animal research

Laboratory animals	NOD.Cg-KitW-41JTyr+PrkdcscidIl2rgtm1Wjl(NBSGW) mice were obtained from Jackson Laboratory (Stock 026622) and used for xenotransplantation experiments. Male and female littermates aged 4-8 weeks were equally distributed across experimental groups. Animals were housed under social conditions (5 mice per cage) with 12 hour/12 hour dark/light cycle and optimal ambient temperature (70F +/- 2F) and humidity (40% +/-10%).
Wild animals	This study did not involve wild animals.
Field-collected samples	This study did not involve field-collected samples
Ethics oversight	The Institutional Animal Care and Use Committee (IACUC) at Boston Children's Hospital approved the study protocol and provided guidance and ethical oversight.

Note that full information on the approval of the study protocol must also be provided in the manuscript.

ChIP-seq

Data deposition

☒ Confirm that both raw and final processed data have been deposited in a public database such as GEO.

☐ Confirm that you have deposited or provided access to graph files (e.g. BED files) for the called peaks.

Data access links<br>*May remain private before publication.*	*For "Initial submission" or "Revised version" documents, provide reviewer access links. For your "Final submission" document, provide a link to the deposited data.*
Files in database submission	*Provide a list of all files available in the database submission.*
Genome browser session<br>(e.g. UCSC)	*Provide a link to an anonymized genome browser session for "Initial submission" and "Revised version" documents only, to enable peer review. Write "no longer applicable" for "Final submission" documents.*

Methodology

Replicates	Three independent biological replicates for each experimental condition were processed separately and the generated libraries were pooled for sequencing
Sequencing depth	ChIP-seq library was quantified with Agilent Bioanalyzer. The libraries were sequenced at Broad Institute Genomic Services by using the Illumina NextSeq 500 platform and the 150-bp paired-end configuration to obtain at least 30 million reads per sample.
Antibodies	anti-CTCF (abcam, ab128873, RRID:AB_11144295)<br>anti-IgG (Diagenode, C15410206, RRID: AB_2722554)
Peak calling parameters	*Specify the command line program and parameters used for read mapping and peak calling, including the ChIP, control and index files used.*
Data quality	*Describe the methods used to ensure data quality in full detail, including how many peaks are at FDR 5% and above 5-fold enrichment.*
Software	*Describe the software used to collect and analyze the ChIP-seq data. For custom code that has been deposited into a community repository, provide accession details.*

Flow Cytometry

Plots

Confirm that:

☒ The axis labels state the marker and fluorochrome used (e.g. CD4-FITC).

☒ The axis scales are clearly visible. Include numbers along axes only for bottom left plot of group (a 'group' is an analysis of identical markers).

☒ All plots are contour plots with outliers or pseudocolor plots.

☒ A numerical value for number of cells or percentage (with statistics) is provided.

Methodology

Sample preparation	Adult mobilized peripheral blood CD34+ stem and progenitor cells or umbilical cord-derived CD34+ stem and progenitors were cultured in StemSpan II with cc100 cocktail, TPO at 100ng/ml, and UM171 (35nM) and analyzed at the indicated days.
Instrument	Becton Dickinson (BD) LSRII<br>Becton Dickinson (BD) LSR Fortessa<br>Becton Dickinson (BD) Accuri C6
Software	FlowJo software (v.10.6)
Cell population abundance	Abundance post-sort (purity check) was not measured due to low frequency of LT-HSCs
Gating strategy	FSC-A/SSC-A; FSC-A/FSC-W; SSC-A/SSC-W; CD34+CD45RA-CD133+EPCR+ITGA3+. Fluorescence minus one (FMO) controls were used to define the boundaries between positive and negative signals.

☒ Tick this box to confirm that a figure exemplifying the gating strategy is provided in the Supplementary Information.
